# Population Phylogenomics and Genetic Structure of the Polyphagous Leafminer, *Liriomyza trifolii* (Burgess) (Diptera: Agromyzidae)

**DOI:** 10.1111/eva.70132

**Published:** 2025-07-09

**Authors:** Jing‐Li Xuan, Sonja J. Scheffer, John Soghigian, Brian Cassel, Matthew L. Lewis, Shu‐Peng Li, Jian‐Yang Guo, Ravindra C. Joshi, Wan‐Xue Liu, Brian M. Wiegmann

**Affiliations:** ^1^ Anhui Engineering Research Center for Green Production Technology of Drought Grain Crops, College of Life Sciences Huaibei Normal University Huaibei People's Republic of China; ^2^ Department of Entomology and Plant Pathology North Carolina State University Raleigh North Carolina USA; ^3^ State Key Laboratory for Biology of Plant Diseases and Insect Pests, Institute of Plant Protection Chinese Academy of Agricultural Sciences Beijing People's Republic of China; ^4^ Anhui Province Key Laboratory of Pollutant Sensitive Materials and Environmental Remediation, College of Life Sciences Huaibei Normal University Huaibei People's Republic of China; ^5^ Systematic Entomology Laboratory USDA‐ARS Beltsville Maryland USA; ^6^ Department of Comparative Biology and Experimental Medicine University of Calgary Calgary Alberta Canada; ^7^ Anhui Watermelon and Melon Biological Breeding Engineering Research Center, College of Life Sciences Huaibei Normal University Huaibei China; ^8^ Philippine Rice Research Institute, Maligaya Science City of Muñoz Nueva Ecija Philippines

**Keywords:** anchored phylogenomics, cryptic species, genetic structure, invasive species, *Liriomyza trifolii*, phylogeography

## Abstract

The agromyzid leafminer *Liriomyza trifolii* (Burgess) is an important polyphagous pest of vegetable crops and ornamental plants. It is native to the Americas but has spread throughout the world over the past 50 years. Previous molecular research has indicated that this species contains highly distinct mitochondrial lineages suggestive of cryptic species. To better interpret the mitochondrial divergence, we used anchored hybrid enrichment datasets in order to conduct genome‐wide phylogenetic analyses. We found that individuals of *L. trifolii* from pepper and tomatillo populations form a monophyletic group (“PT group”) distinct from the remaining *L. trifolii* (“non‐PT group”). These results corroborate previous mitochondrial and nuclear datasets and indicate an absence of gene flow between the PT and non‐PT groups. This is consistent with previous work on reproductive isolation and oviposition preferences, and provides substantial evidence that the PT group represents a distinct and previously unrecognized species. The presence of two species within a nominally single pest species has important implications for management. Although there was only weak genetic differentiation between geographically disparate groups of non‐PT *L. trifolii* , a monophyletic group of Chinese specimens was found in a coalescent‐based analysis that is concordant with the history of invasions in Asia. Our study provides important new insight into geographic and host‐associated structure in *L. trifolii* .

## 1 Introduction

A variety of factors contribute to genetic divergence among natural populations, including geographic isolation, selection on ecological differences, and responses to environmental or climatic conditions (Sexton et al. 2014; Frévol et al. 2023; McGreevy et al. 2024). In recent decades, detailed investigations of ecological specialization in herbivorous insects have shown that adaptation to host plants can play a significant role in the process of genetic differentiation and diversification, even in sympatry (Orsucci et al. 2016; Bouzas et al. 2021; Hinojosa et al. 2023). Such ecological isolation or host‐associated differentiation (HAD) is considered a classic model of ecology‐mediated selection (Stireman III et al. 2005; Heard 2012; Leung and Beukeboom 2022), and this process can result in the formation of genetically distinct host‐associated lineages or host races, or even sibling species (Nosil and Crespi 2006; Poveda‐Martínez et al. 2020). Previous studies have demonstrated that host‐associated differentiation may play an important role in generating the remarkable diversity of phytophagous insects at both macroevolutionary and microevolutionary scales (Razmjou et al. 2010; Forbes et al. 2017; Hinojosa et al. 2023).

A number of agriculturally important pest species contain host‐associated populations and cryptic species (Scheffer and Lewis 2001; Simmons and Scheffer 2004; Correa et al. 2023). In the case of polyphagous species, the multitude of host plants used in concert with high population abundances may conceal distinct host‐associated lineages within what appears to be a single species (Scheffer and Lewis 2006; Hendrichs et al. 2015). It is important that these lineages are recognized because genetically distinct populations of pest species may differ in significant characteristics such as host use, insecticide resistance, thermal tolerances, and susceptibility to parasitoids. Knowledge of such differences is crucial for the design of predictive and effective control methods (Correa et al. 2023; Wang et al. 2023).

The leaf‐mining fly *Liriomyza trifolii* (Burgess) (Diptera: Agromyzidae) is a highly damaging and broadly polyphagous pest species. It has been recorded from at least 400 plant species across more than 40 plant families, including dozens of agriculturally important crops (Minkenberg and Van Lenteren 1986; Spencer 1990; Xuan et al. 2023). Major vegetable crops include beans, celery, lettuce, onion, peas, peppers, squash, and tomato, as well as ornamental crops such as chrysanthemum, gerbera, and gypsophila (Spencer 1973; Minkenberg and Van Lenteren 1986). As *L. trifolii* moves into new regions, it continues to expand its diet by colonizing novel local hosts not previously experienced (Parrella and Keil 1984; Spencer 1990), consistent with its polyphagous nature. However, the number, as well as the taxonomic and chemical diversity, of *L. trifolii* 's host plants provides substantial opportunities for selection for dietary specialization that might lead to the formation of host races and cryptic species.

*Liriomyza trifolii* was originally described from Washington, D.C., and until the 1970s was only known from eastern North America and Venezuela (Spencer 1973; Parrella and Keil 1984; Spencer and Steyskal 1986). During the 1970s, *L. trifolii* spread from Florida to California and possibly other regions within the United States (Parrella and Keil 1984; Reitz et al. 2013). In addition, interception records from that period also implicate Florida as the source of early global invasions to Colombia, Denmark, Israel, Kenya, the Netherlands, Sweden, and the UK (Spencer 1965; Minkenberg and Van Lenteren 1986; Minkenberg 1988). *L. trifolii* has continued to spread, most recently to China in 2005 and Australia in 2021 (Maino et al. 2023), although this spread is unlikely to have been directly from Florida and instead probably represents regional expansion from other colonized locations. Following its arrival in China, it rapidly spread from Guangdong Province to most provinces across south‐east, coastal, and central China in only a few years (Gao et al. 2017).

Phylogeographic and population genetic studies have assessed host use, genetic structure, and global invasions by *L. trifolii* . Significant differences in RFLP (restriction fragment length polymorphism) frequencies were found between *L. trifolii* populations from peppers compared to populations from other hosts in California (Morgan et al. 2000). Using global samples, analysis of mitochondrial DNA sequence data uncovered highly diverged mitochondrial clades “A” and “W” suggesting the presence of cryptic species (Scheffer and Lewis 2006; Pérez‐Alquicira et al. 2019). In this work, 47 specimens from peppers, collected in California, Florida, Honduras, and Mexico, formed a shallow but monophyletic “pepper” clade within mitochondrial clade “W,” indicative of a host race or cryptic species. None of the 131 specimens associated with other hosts from disparate locations, including Arizona, California, Florida, New York, Israel, Italy, the Philippines, and South Africa, belonged to the pepper clade (Scheffer and Lewis 2006). Subsequent research from multiple collections in Mexico corroborated the “pepper” clade and found that specimens from tomatillos also belong in this group (Pérez‐Alquicira et al. 2019). Analysis of *L. trifolii* from multiple hosts in China uncovered no additional host‐associated groups (Chen et al. 2019).

As of 2006, introduced *L. trifolii* populations across globally disparate Old World and Asian locations were almost entirely composed of a single mitochondrial haplotype in group W (Scheffer and Lewis 2006; Scheffer et al. 2006). The general paucity of mitochondrial haplotypes in globally introduced *L. trifolii* populations has been corroborated from densely sampled populations in the Philippines, China, and Australia (Scheffer et al. 2006; Chen et al. 2019; Xu et al. 2021).

Here, we use genomic data from target capture collected using anchored hybrid enrichment (AHE) and single nucleotide polymorphisms (SNPs) called from a draft genome of *L. trifolii* (Vicoso and Bachtrog 2015) to further confirm and re‐evaluate patterns of genetic variation associated with host plant use and geography in this invasive species. Specifically, we ask: (1) Are the deep mitochondrial lineages A and W that were detected in previous studies of *L. trifolii* corroborated by genomic data, (2) Are additional host‐specialized lineages suggestive of HAD present within *L. trifolii* , (3) Can our phylogenomic data reveal fine‐scale phylogenetic relationships among populations of *L. trifolii* , and (4) Does the global population structure of *L. trifolii* allow the determination of possible source(s) of invasive populations?

## 2 Materials and Methods

### 2.1 Taxon Sampling and Target Enrichment Data Collection

In this study, 171 individual specimens (169 *L. trifolii* + 2 *L. sativae* Blanchard) were sampled from 23 host plant crops across 10 families and from at least 47 collection sites in 12 countries throughout the New and Old Worlds (Table S1). These samples were collected primarily by rearing specimens from mined leaves and conducting targeted sweep netting. Specimens were killed in 95% ethanol and stored in a −80°C freezer. Some specimens obtained by other scientists had been collected and stored in a range of ethanol concentrations (70%–95%) and switched to 95% ethanol upon arrival in the Scheffer lab.

Whole genomic DNA was extracted with the QIAGEN DNeasy Blood & Tissue Kits (QIAGEN Inc., Hilden, Germany) following the protocol from the manufacturer, except for the final step where the genomic DNA was eluted twice with 30 μL of the AE buffer. Whole genome amplifications were carried out with the REPLI‐g Mini Kit (QIAGEN Inc., Hilden, Germany) following the manufacturer's protocol. To prepare genomic templates for anchored hybrid enrichment (AHE), whole genomic DNAs were sheared to a fragment size of 300 base pairs (bp) on a Covaris S220/E220 Focused‐ultrasonicator (Covaris Inc., Massachusetts, USA). Sheared DNAs were used for library preparation.

Sequencing was performed on an Illumina NovaSeq 6000 sequencer with paired‐end raw reads, or for a small number of samples by single‐read sequencing on an Illumina HiSeq 2500 platform. The genomic raw reads used in this study were submitted to the Sequence Read Archive (SRA) PRJNA1201066 at the National Center for Biotechnology Information (NCBI) database. Raw reads were trimmed with default parameters using the package FASTP (Chen et al. 2018). De novo assembly was carried out using trimmed reads in TRINITY v.2.2 (Grabherr et al. 2011). Orthology prediction was conducted using a graph‐based, reciprocal blast approach in OrthoGraph v.0.6.1 (Petersen et al. 2017). Single‐copy orthologous genes were verified as top hits for Diptera genes using the BLAST program (Altschul et al. 1990). Sequences were aligned with the L‐INS‐i algorithm (−localpair, −addfragments, and ‐maxiterate 1000 flags) using MAFFT v.7.481 (Katoh and Standley 2013). Ambiguously or erroneously aligned regions or sections were removed from alignments using AliCUT (Kück et al. 2010). Nucleotide supermatrices were concatenated using FASCONCAT‐G (Kück and Longo 2014) and were used to conduct phylogenetic analyses.

### 2.2 Single Nucleotide Polymorphisms (SNPs) Extraction

Trimmed sequencing reads were individually aligned/mapped into draft *L. trifolii* reference genome downloaded from NCBI (GenBank: JXHJ00000000.1) using the BWA (Burrows–Wheeler Aligner) program (Li and Durbin 2009). Output SAM (sequence alignment map) format files were converted to BAM (binary alignment map) format using “samtools view” command with the ‐bS option, and then the BAM files were sorted with “samtools sort” command such that the alignments occurred in predicted genomic order (Li et al. 2009). The mapping quality of sorted BAM files were evaluated in QualiMap with bamqc command (García‐Alcalde et al. 2012), which provides an overall insight of the alignment quality and is useful for sample selections for further analysis. Samples with more than 30% mapped reads were used for downstream variant selections.

Mapped and unmapped BAM files for each sample were merged using Picard's MergeBamAlignment tool with default parameter settings except for the parameter “PAIRED_RUN,” specifically, “true” setting for samples with pair‐end reads and “false” for few samples with single‐end reads. Duplicates in BAM files were marked with default parameters using Picard's MarkDuplicates tool. Genetic variants were called for each sample over alignments generated by mapping to the reference genome of *L. trifolii* using the Genome Analysis Toolkit (GATK) HaplotypeCaller with the ploidy parameter setting to two. SNP variants were selected with the command “‐‐select‐type‐to‐include SNP” using GATK's SelectVariants tool, which separately generates a variant calling format (vcf) file for each sample (Brouard et al. 2019; Van der Auwera and O'Connor 2020). The number of original variant sites in each vcf file were checked with the command “grep ‐v ‘#’.” Samples were filtered based on the threshold of the number of original variant sites. Specifically, samples including more than 91,000 variant sites were kept for downstream variant filtering process (Table S2). SNP variants per sample were filtered with the filter commands (−‐select‐expressions “QUAL > = 20.0 && DP > = 10.0”) based on quality score and read depth conducted in GATK's SelectVariants tool.

These filtered vcf files were merged into a single vcf file using the “bcftools merge” command. The resulting vcf file was filtered using the filter command with the following criteria “‐‐max‐missing 0.80 ‐‐minQ 20—minDP 10 ‐‐maf 0.02” flags implemented in VCFtools (Danecek et al. 2011). SNP variant filtering based on Hardy–Weinberg equilibrium (HWE) was not performed since HWE deviations might have occurred in some of the studied populations due to genetic drift (Bhuiyan et al. 2021). To detect pairwise relatedness, the relatedness statistic among the samples was estimated using the final filtered vcf file, and samples with a RELATEDNESS_PHI value > 0.125 suggesting parent‐offspring or siblings were removed using VCFtools. The filtered SNPs were used to conduct downstream population genetic analysis.

### 2.3 Phylogenetic Analysis

For phylogenetic analyses, two types of concatenated nucleotide supermatrices were constructed: (I) a concatenated nucleotide supermatrix with 169 individuals of *L. trifolii* ; (II) a concatenated supermatrix including 126 *L. trifolii* representative individuals with higher quality of sequencing data. In both concatenated supermatrices, two *L. sativae* individuals, the sister species to *L. trifolii* , were included as outgroups to root the phylogenetic trees (Xuan et al. 2023).

Maximum likelihood (ML) trees were conducted with the best‐fit model selection in ModelFinder under the Bayesian Information Criterion (BIC) using the command “‐TESTNEW” implemented in IQ‐TREE 2.1.2 (Kalyaanamoorthy et al. 2017; Minh et al. 2020). Support values on the nodes of the trees were obtained by 1000 replicates of Ultrafast bootstrap approximation (Minh et al. 2013). Mitochondrial clade, collection location, and host plant family were mapped on the ML phylogeny consisting of 128 individuals using the online mapping tool, Interactive Tree Of Life (iTOL) v.4 (Letunic and Bork 2019).

A coalescent species tree was reconstructed using gene trees with 128 exemplars implemented in the program ASTRAL‐III (Zhang et al. 2018). These gene trees were generated using nucleotide loci over 400 bp in length under the GTR model in IQ‐TREE. A phylogenetic network from nucleotide alignments from 128 individuals was computed using uncorrected‐*p* distances inferred from the program SplitsTree4 (Huson and Bryant 2006). A neighbor‐joining (NJ) tree was also constructed using nucleotide alignments in the program SplitsTree4.

### 2.4 Genetic Structure Analysis

Principal Components Analysis (PCA) was performed to identify population genetic structure using Plink v.1.9 (Purcell et al. 2007). Plink format files were generated using a filtered SNP vcf file from 109 *L. trifolii* individuals. Input for Plink was carried out with the “‐‐make‐bed” command option. Subsequently, the outputs were used as inputs to generate eigenvalues and eigenvectors within “‐‐pca” command on Plink. To better explain the contribution of each component to genetic variations, we transformed each principal component into the percentage of total genetic variance, and all percentages of genetic variance were summed to 100%. Percentage genetic variance for each component was viewed by bar charts, and the scatterplot of the first two main principal components (PC1 and PC2) was conducted using R packages tidyverse and ggplot2 in RStudio. Estimates of individual admixture/ancestry coefficients within a genotype matrix were also conducted using R function snmf in package LEA performed in RStudio (Frichot et al. 2014; Frichot and François 2015). To avoid stochastic effects from a single analysis, 10 iterations at each value of potential genotype cluster (*K*) were conducted.

Parameter weighted *Fst* (fixation index) was used to measure pairwise genetic differentiation between host‐associated populations based on the Weir and Cockerham approach (Weir and Cockerham 1984). These were calculated using a non‐overlapping 500 bp sliding window across the genome and implemented in VCFtools (Danecek et al. 2011). We used 133 *L. trifolii* individuals across 10 plant populations with a sample size equal to or greater than four to analyze *Fst* using the filtered SNPs. A heatmap was constructed using mean *Fst* values between host plant populations using GraphPad Prism 10. We also calculated *Fst* values for samples from three countries having the largest sample sizes: United States, China, and Philippines. These represent both native (United States) and introduced populations (China and Philippines). Three analyses of molecular variance (AMOVA) for host‐associated and geographically separated populations were conducted with less than 0.05 missing data implemented in Arlequin v.3.5.2.2 (Excoffier and Lischer 2010). The Arlequin input file was translated from the vcf SNP file using program PGDSpider v.2.1.1.5 (Lischer and Excoffier 2012). Standard AMOVA computations were applied with 1000 permutations at the significance level of 0.05.

## 3 Results

### 3.1 Phylogenetic Analyses

Two distinct concatenated sequence supermatrices were generated: (I) a concatenated supermatrix with 232,815 nucleotide positions from 390 orthologous single‐copy nuclear genes in 171 individual specimens, and (II) a concatenated supermatrix with 269,448 nucleotide characters across 428 nuclear orthologs in 128 representative individuals. The information content contained within each nucleotide locus of over 400 bp in length is shown in Table S3.

**FIGURE 1:**
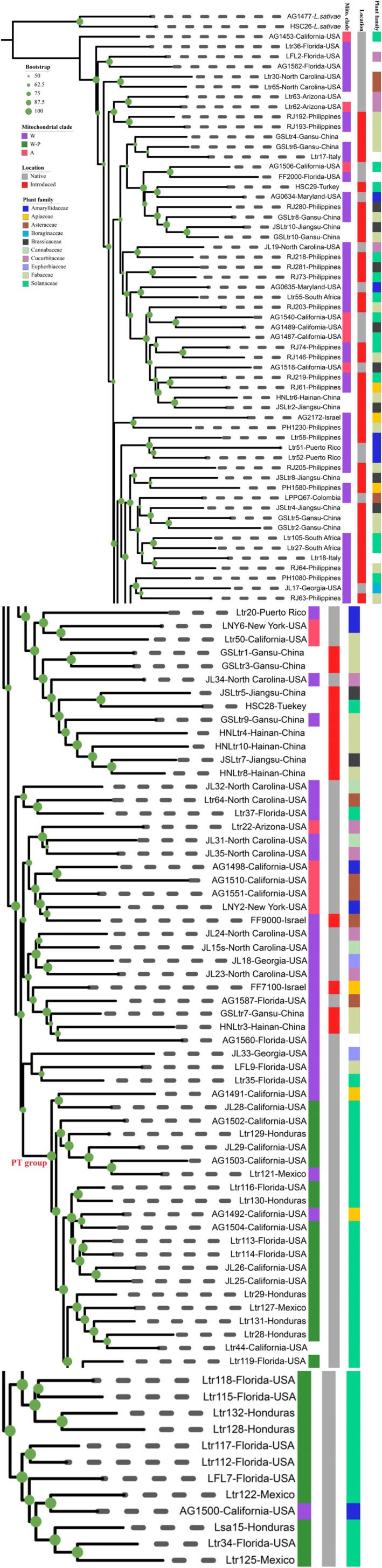
Maximum likelihood phylogeny of *Liriomyza trifolii* estimated from anchored enrichment alignments for 128 individual samples conducted in IQ‐TREE. Ultrafast Bootstrapping (UFBoot) values above 50% are indicated by green dots on the nodes of the phylogenetic tree. Tips on the tree are shown sample code and collection location. The columns from inner to outmost orderly represent the mitochondrial clade (A, W‐P, W), location (native or introduced), and host plant family. Individuals from America are considered to be native, and others are recorded to be introduced.

**FIGURE 2:**
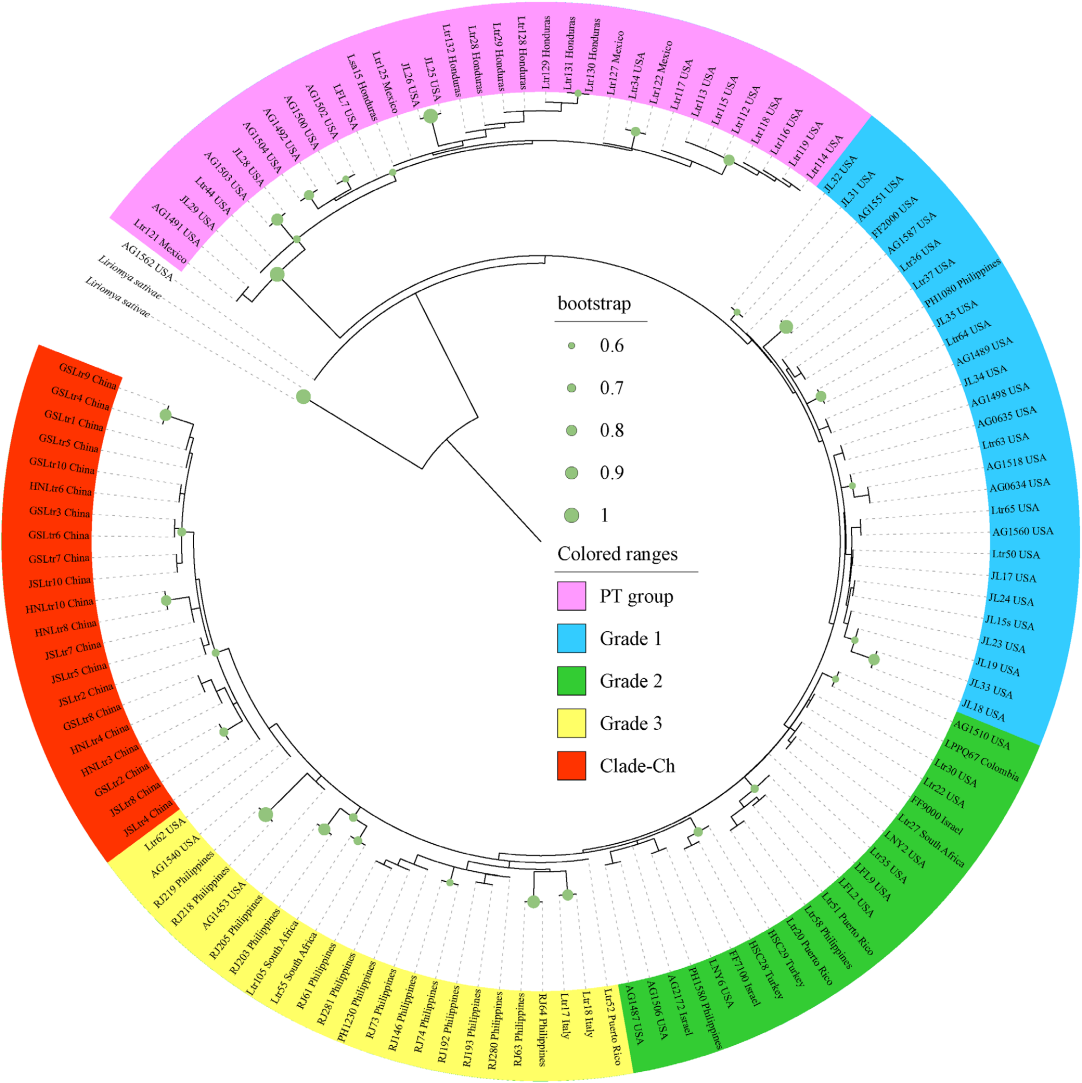
Coalescent species tree performed in ASTRAL‐III. Pepper clade/species colored by pink is distinct but slightly inside non‐pepper group. All the Chinese samples form a monophyletic group at the tip. Three “grades” show interesting patterns‐blue grade is almost (26/27) entirely United States; green grade is a mix of United States (47%) and global samples with only 2/21 from Asia (the Philippines); yellow grade is dominated by the Philippines with a few global and a few US samples. Clade red is all Chinese specimens (Clade‐CH).

All the phylogenetic trees constructed in this study found a distinct and well‐supported clade consisting of all pepper and tomatillo specimens, as well as three individuals swept from onion and celery (Figures 1 and 2, Figures S1–S3). This pepper‐tomatillo clade (“PT group”) was present only in the Americas, including California, Florida, Mexico, and Honduras. The remaining individuals of *L. trifolii* formed a larger “non‐PT group” comprising interspersed specimens belonging to the highly divergent mitochondrial “A” and “W” clades found in previous analyses (Scheffer and Lewis 2006). This group comprised diverse populations sampled broadly from many host plants and locations, both native and introduced (Figures 1 and 2, Figure S1). There was no evidence of any additional host‐restricted groups beyond the PT clade.

**FIGURE 3:**
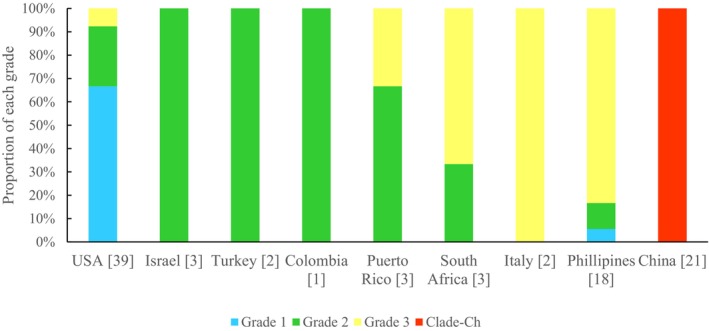
Histogram of the geographic distribution of the grades and Chinese clade from coalescent tree in Figure 2.

In addition, the coalescent analysis recovered considerable geographic signal in the non‐PT *L. trifolii* . There was a shallow but distinct monophyletic clade containing all specimens collected from China (Figure 2). In addition, three grades were present that progressed in a rough pattern of timing and locations to that of invasions by *L. trifolii* . The first grade (blue) is composed almost entirely of native and North American specimens (96%) (Figures 2 and 3). The second grade (green) includes both North American (47%) and global (53%) locations. Only 10% of these global specimens are from Asian locations. The third grade is more than half Asian (the Philippines) mixed with other United States and early introduced locations. The monophyletic Chinese clade emerges from this third grade (Figures 2 and 3).

### 3.2 Population Genetic Analyses

**FIGURE 4:**
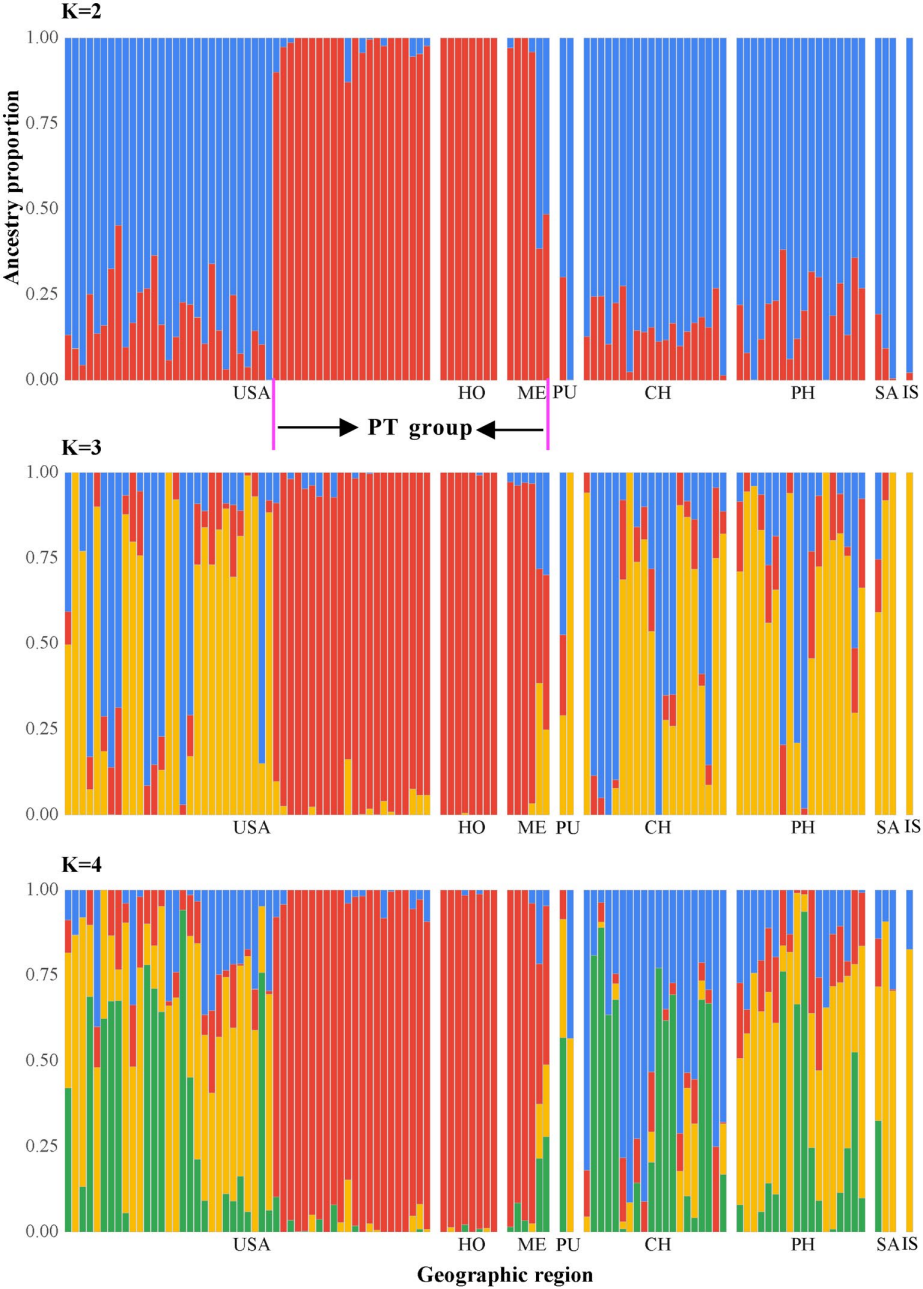
Admixture plot of ancestry proportion estimates at *K* = 2–4. X axis represents eight geographic populations, while Y axis indicates the ancestry proportion. Each narrow vertical bar represents one individual. The abbreviations in geographic regions are as follows: CH, China; HO, Honduras; IS, Israel; ME, Mexico; PH, Philippines; PU, Puerto Rico; SA, South Africa; USA, United States of America. Individuals marked by PT group represent the same ones on the phylogeny.

A total of 915 SNPs were obtained for 109 individuals collected from eight countries and at least 17 plant hosts. Admixture analysis identified the pepper and tomatillo clade from the United States (California and Florida), Honduras, and Mexico as distinct from the other samples regardless of which value of *K* = 2–4 (Figure 4).

**FIGURE 5:**
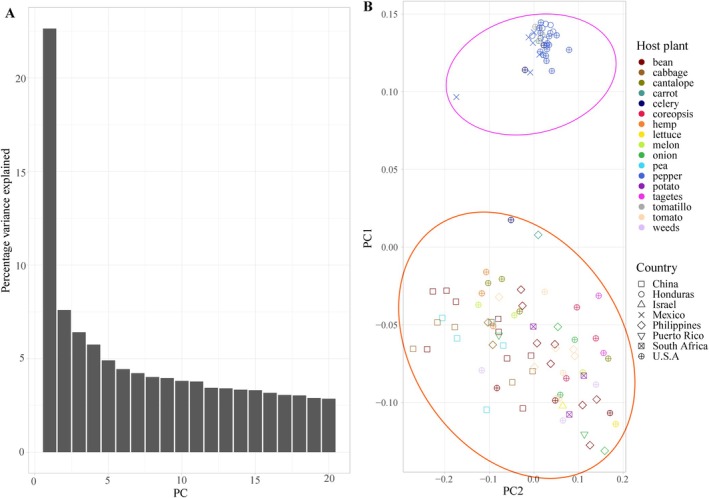
Genetic variance in SNPs sampled for *Liriomyza trifolii* . (A) Fraction of observed genetic variance contained in each principal component; (B) scatterplot of the first two major principal components from principal component analysis (PCA) analysis. Shape symbols indicate geographic populations from distinct countries, and color indicates host‐plant population. The top cluster circled by pink represents the “PT group,” while bottom cluster circled by orange is the “non‐PT group.”

**FIGURE 6:**
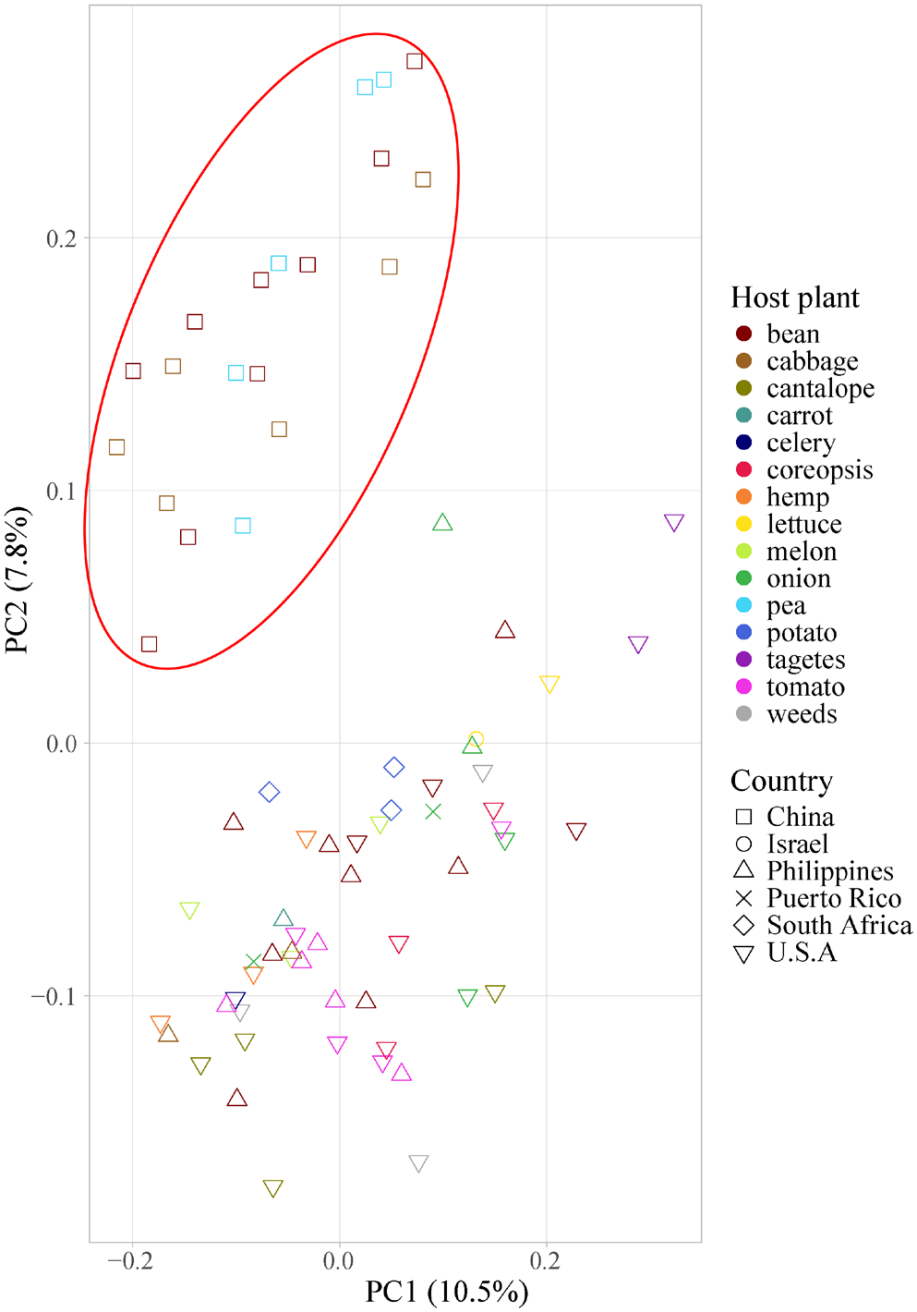
PCA plot with PT group excluded. One separate genetic cluster is associated with Chinese populations circled in red on the PCA plot.

In the principal component analysis, the percentage of genetic variance explained ranged from 2.9 to 22.6 (Figure 5A), and two highly distinct clusters corresponding to the PT and non‐PT groups were identified (Figure 5B). Within the non‐PT group, no additional clusters by host plant or location were found. In addition, individuals from broadly sympatric, primarily the same countries, are not clustered together on the PCA plots (Figure 5B). After removing the PT group samples, a second PCA analysis found a distinct cluster exclusively comprised of individuals from China (Figure 6).

**FIGURE 7:**
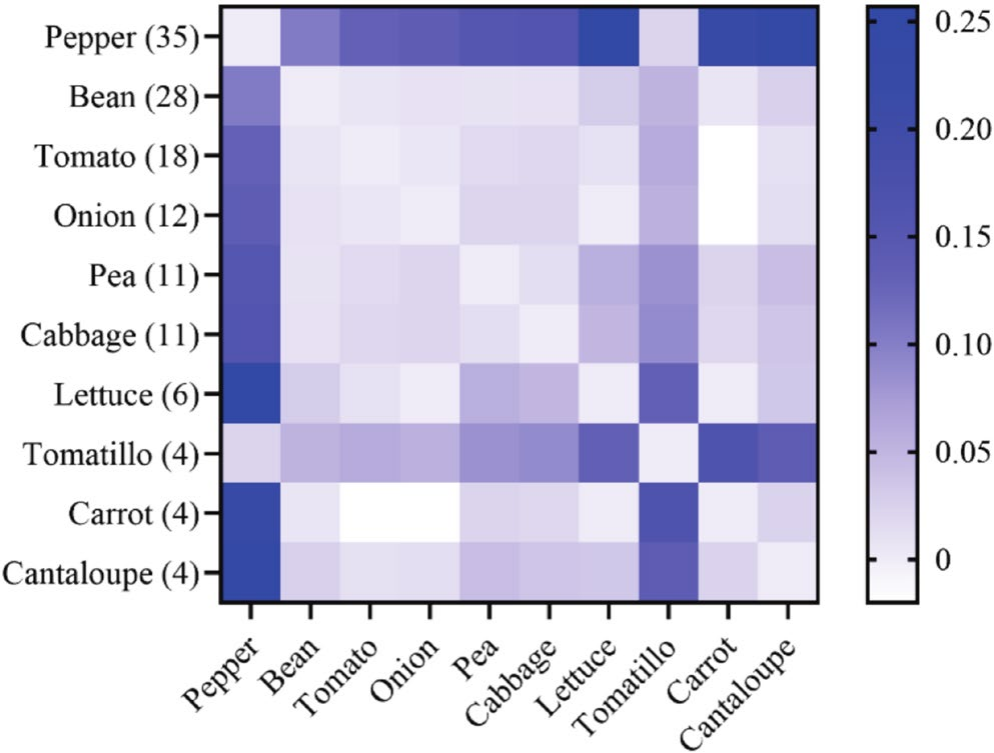
A heatmap of mean *Fst* estimate for pairwise genetic differentiation between host plant populations. The value in the parentheses represents the sample size for a host‐plant population.

We found significant moderate‐ or high‐level *Fst* values for genetic differentiation between pepper‐associated flies and those feeding on any other host plant, with the exception of the tomatillo‐feeding population (Figure 7). Low genetic differentiation was found among pooled samples comprising China, the Philippines, and the USA populations (Table S4). Based on AMOVA analysis, significant differences were found among populations and within populations in host plant and geography (Table S5), and between the PT group and other non‐PT hosts (Table S6).

## 4 Discussion

*Liromyza trifolii* is one of several highly polyphagous pests of vegetable and horticulture crops (Spencer 1973, 1990). This is a small species having morphological characters that are similar to both other agricultural pests as well as to other species in the large genus *Liriomyza* (Spencer and Steyskal 1986; Lonsdale 2011). These similarities as well as essentially no species‐level diagnostic larval or pupal characters have made *Liriomyza* taxonomically challenging. For example, there are at least four formal synonyms associated with *L. trifolii* as well as multiple (English) common names (Spencer 1973). Difficult species identification and challenging risk assessment of *L. trifolii* due to morphological similarity with closely related *Liriomyza* species have facilitated the global spread and establishment of this species (Parrella and Keil 1984; Morgan et al. 2000; Scheffer and Lewis 2006; Scheffer et al. 2006). Morphological homogeneity could also obscure the presence of cryptic species or lineages.

The highly diverged mitochondrial clades “A” and “W” identified in previous studies did not form monophyletic groups in any of the analyses using nuclear sequence or SNP data. Instead, these formed a single clade (non‐PT) having members of both groups interspersed. A number of factors can cause a mismatch between mitochondrial and nuclear data, including infection by *Wolbachia*, introgressive hybridization, incomplete lineage sorting, and coalescence of previously diverged lineages, also known as “reverse speciation” (Gompert et al. 2008; Rodriguero et al. 2010; Jiang et al. 2018; Kearns et al. 2018; Poroshina et al. 2020; Zadra et al. 2021). Although we do not know the cause of the previously discovered deep mitochondrial divergence of A and W within *L. trifolii* , it is striking that populations from Florida, the eastern United States, and all global invasive populations belonged to the mitochondrial W clade, which is consistent with the original source of many invasive populations being Florida (Parrella 1982; Scheffer and Lewis 2006). Most of the samples from California belonged to the A clade, as well as specimens from Arizona and New York (a greenhouse population likely a result of an importation from California). This difference was observed despite the previous well‐accepted evidence that populations in California are the result of introductions from Florida (Parrella 1982; Reitz and Trumble 2002).

Analysis of the non‐PT *L. trifolii* did not uncover any additional host‐associated groups, but the *Fst* and AMOVA results indicate some genetic differentiation by host plant and/or location. Numerous studies prior to 1970 indicated some degree of genetic variation in host‐use within pest *Liriomyza*, including differences in host‐associated mortality. Unfortunately, the rampant confusion in species identifications at that time precludes tying the studies to specific *Liriomyza* species as currently recognized (Spencer 1973). However, that such differences have been observed within polyphagous *Liriomyza* species raises the possibility that local adaptation in host preference and host‐associated performance could result in subtle genetic differences only detectable with rapidly evolving markers as seen in our data. The evolution of insecticide resistance in *L. trifolii* (see below) can occur in as little as 2 years (Parrella et al. 1984; Sanderson et al. 1989; Ferguson 2004). Similarly, once selection is relaxed, insecticide resistance can be lost in only several generations (Parrella and Trumble 1989; Ferguson 2004). This raises the possibility that short‐term adaptation to plant hosts could occur in this species only to be lost as ecological conditions and cropping systems change. Further exploration will require a greater degree of sample replication of populations from hosts in sympatry and in allopatry.

In all analyses, we found evidence of a distinct pepper and tomatillo‐associated group consistent with previous mitochondrial findings. Our samples in the PT group originated in California, Florida, Honduras, and Mexico. They consist of all pepper and tomatillo samples, as well as three specimens swept from celery and onion. The host‐association data of these three specimens cannot be considered definitive, as they were obtained by sweep‐netting in fields that also contained commercial pepper crops. The other specimens swept from celery and onion in the same fields all came out in the non‐PT clade. The PT clade was distinct from the remaining non‐PT *L. trifolii* in both phylogenomic and SNP analyses, despite broad sympatry of several collections in Florida and California (Figures 1, 2, 4, and 5, Figure S2).

A feature of host‐associated divergence and speciation in many phytophagous insects appears tied to host use involving female oviposition preferences and larval growth/mortality across different host plants (Via 2001; Bolnick and Fitzpatrick 2007). In addition to the correspondence in molecular data, further support that the PT and non‐PT groups are biologically distinct comes from behavioral studies showing substantial reproductive isolation between flies in the two groups. Both PT and non‐PT flies prefer to mate with flies from the same clade, and PT flies only mate while exposed to pepper (Reitz and Trumble 2002: “central” population is PT, while “southern” population is non‐PT, see Scheffer and Lewis 2006). In addition, several studies have found that pepper foliage or its chemical extracts are strong, and at times, perfect deterrents to oviposition on normally acceptable host plants by non‐PT females (Kashiwagi, Horibata, et al. 2005, Kashiwagi, Mikagi, et al. 2005; Dekebo et al. 2007; Tebayashi et al. 2007).

Together, the results of this study, along with those of the earlier studies discussed here, provide strong evidence that the PT and non‐PT groups are essentially functioning as separate species. Concordance of our phylogenomic datasets of multilocus nuclear sequences and thousands of SNPs with previous mitochondrial datasets showing divergence between PT and non‐PT populations indicates an absence of nuclear or mitochondrial gene flow between these groups. In addition to the behavioral evidence referenced above supporting reproductive isolation, an absence of gene flow can be seen by the small number of mixed ancestry pepper flies in the admixture plot (Figure 4).

Recognition of the PT clade as a distinct species is important for understanding basic biological differences relating to pest management. Genetically isolated species have different traits and are on independent evolutionary trajectories. Traits such as oviposition behavior, host‐associated mortality, life cycle, insecticide and parasitoid susceptibilities often differ between species in substantial ways. Greater precision understanding of how biological traits differ as well as how they affect population dynamics could offer new approaches to managing this species. For example, *L. trifolii* from peppers differs from non‐pepper flies in susceptibility to ionizing irradiation in phytosanitary treatments associated with quarantine (Hallman et al. 2011), potentially of great importance to managing invasion risks involving infested commodities at ports of entry.

Most obviously, high abundances on peppers will not necessarily mean high abundances on other crops, and vice versa. The second author (S.J.S.) has been in a large vegetable field in Florida where the leaves of peppers were extremely heavily mined, and piles of pupae lay beneath the plants. Other plant crops in the field had almost no mines even when planted only 5 m away. Similar observations have been made elsewhere: in Jiangsu Province, China, the invasive and abundant *L. trifolii* rarely mines pepper, and the survival of any mines formed on those plants is low (pers. comm., Dr. Du Yu‐Zhou, Yangzhou University).

Knowledge that the PT and non‐PT groups are distinct may provide critical information for the management of insecticide resistance, particularly with respect to IPM where thresholds based on abundance or levels of damage are used to trigger insecticide applications. For example, indiscriminate spraying of all vegetable crops when only peppers or tomatillos exhibit high levels of damage is economically and environmentally costly. In addition, it has been well documented that overuse of insecticides can trigger the rapid evolution of insecticide resistance in the pest *Liriomyza*, leading to highly damaging outbreaks (Parrella et al. 1984; Sanderson et al. 1989). At one point, the too‐frequent application of insecticides leading to insecticide resistance resulted in the expected efficacy for any particular compound against *L. trifolii* being only 2 or 3 years (Sanderson et al. 1989; Ferguson 2004). This has resulted in vigorous research on the development and use of insecticides for *L. trifolii* that has spanned 75 years (Sanderson et al. 1989; Gao et al. 2017; Li et al. 2022; Wang et al. 2024).

The PT‐clade may represent a case of host‐associated differentiation that occurred in sympatry, perhaps relating to the distinct chemical profile of *Capsicum*. It has been suggested that host‐associated differentiation can occur very quickly (Bush 1975; Michell et al. 2023). This raises the possibility that the divergence between the PT group and other *L. trifolii* occurred since the arrival of widespread, nonindigenous agriculture in North America. Under such a scenario, the PT clade would have recently evolved from the non‐PT clade within the past few hundred years. In the ML phylogenomic analyses, the PT group was placed well within the remaining, thus paraphyletic, non‐PT *L. trifolii* (Figure 1) corroborating previous mitochondrial results (Scheffer and Lewis 2006). The coalescence analysis here also found non‐PT *L. trifolii* to be paraphyletic, but in this case, the paraphyly was due to the placement of only a single non‐PT individual (Figure 2).

Alternatively, the PT group could have diverged and adapted to peppers and tomatillos in allopatry over a substantial period of time. Peppers and tomatillos are native to Central and South America where dozens of *Capsicum* species and hundreds of land races occur, with domestication as early as 6000 years ago (Mapes and Basurto 2016; Taitano et al. 2019). A widespread ancestral *L. trifolii* could have become geographically isolated into a southwestern Central/South American population and an eastern North American population. Early records of the distribution of *L. trifolii* are consistent with such a disjunction: eastern North America, Colombia, and Venezuela (Spencer 1973).

Within the native range of North America where structure would be expected, we did not find geographically structured variation in either the phylogenetic or coalescent analysis. However, our samples were primarily from agricultural populations that may share a common source due to frequent shipment between growing regions. In particular, specimens from the geographically distant Florida and California non‐PT group were distributed across the ML phylogenetic trees (Figures 1, S1), consistent with a history of introduction(s) from Florida to California starting in the 1970s (Parrella 1982; Zehnder and Trumble 1984; Reitz and Trumble 2002).

In contrast, the coalescent analysis did uncover a pattern of geographic influence on genetic structure. In this analysis, the non‐PT flies formed three grades of flies from various locations and a monophyletic clade of all specimens from China. That this represents a meaningful group is supported by the PCA analysis, with the PT clade removed, in which the specimens from China were clustered (Figure 6). Although less obvious, the three grades of the coalescent analysis also suggest geographic structure, with the grades moving from the first made up of primarily North American samples, then increasing and comprised of Asian samples (Figures 2 and 3). The final monophyletic clade of specimens from China emerges from the third grade dominated by the Philippines. This might seem to suggest a direct introduction of *L. trifolii* to China from the Philippines, but without extensive sampling from other global locations we cannot draw firm conclusions. However, this is a reasonable hypothesis as the Philippines was one of only a few regions of Southeast Asia to harbor *L. trifolii* prior to its introduction to China (Gao et al. 2017). In any case, our SNP results indicate a genomic approach can detect the history of movement of *L. trifolii* , although geographic sampling will need to be greatly expanded both in terms of locations as well as in sample sizes for robust conclusions.

## 5 Conclusion

Phylogenetic relationships and population genetic structure associated with geographic distribution and host plant use of New‐World species *L. trifolii* were investigated employing anchored phylogenomic and single nucleotide polymorphisms (SNP) datasets. Our results corroborate previous RFLP, behavior, and mitochondrial work indicating that the *L. trifolii* associated with the use of peppers and tomatillos are almost entirely distinct from other *L. trifolii* and represent a previously unrecognized species. In contrast, previously defined deep mitochondrial clades are not monophyletic on our phylogenetic trees and indicate a yet undetermined evolutionary process within *L. trifolii* . Within the non‐pepper/tomatillo group, ML analysis found only weak genetic structure associated with geography or host use. However, the coalescent‐based tree of the non‐pepper/tomatillo group appears to exhibit structure relating to the history of global invasions, particularly with respect to movement through Asia. Our results on the phylogeographic and population genetic structure associated with host use and geography provide important information for improved design of effective management measures and quarantine procedures.

## Ethics Statement

The authors have nothing to report.

## Consent

The authors have nothing to report.

## Conflicts of Interest

The authors declare no conflicts of interest.

## Supporting information


Data S1.

## Data Availability

All raw sequence reads for samples analyzed in this study are available in the NCBI SRA repository (accession: PRJNA1201066).
