# Correction to “Population Phylogenomics and Genetic Structure of the Polyphagous Leafminer, *Liriomyza trifolii* (Burgess) (Diptera: Agromyzidae)”

**DOI:** 10.1111/eva.70175

**Published:** 2025-11-03

Jing‐Li Xuan, Sonja J. Scheffer, John Soghigian, Brian Cassel, Matthew L. Lewis, Shu‐Peng Li, Jian‐Yang Guo, Wan‐Xue Liu, Brian M. Wiegmann. 2025. Population Phylogenomics and Genetic Structure of the Polyphagous Leafminer, *Liriomyza trifolii* (Burgess) (Diptera: Agromyzidae). *Evolutionary Applications* 18, no. 7: e70132.

An author, Dr. Ravindra C. Joshi, was omitted from the author list in the published version. This author provided valuable specimens used in this study, who should be in the author list. Therefore, the correct author list should be “Jing‐Li Xuan, Sonja J. Scheffer, John Soghigian, Brian Cassel, Matthew L. Lewis, Shu‐Peng Li, Jian‐Yang Guo, Ravindra C. Joshi, Wan‐Xue Liu, Brian M. Wiegmann.”

We apologize for this author omission.

