# Supplementary material for: Population Phylogenomics and Genetic Structure of the Polyphagous Leafminer, Liriomyza trifolii (Burgess) (Diptera: Agromyzidae)
Source: Evol Appl. 2025 Jul 9;18(7):e70132. doi: 10.1111/eva.70132 (PMC12241706; doi:10.1111/eva.70132)
Supplement: Supplementary file 1 — Data S1. [file EVA-18-e70132-s001.docx]

**
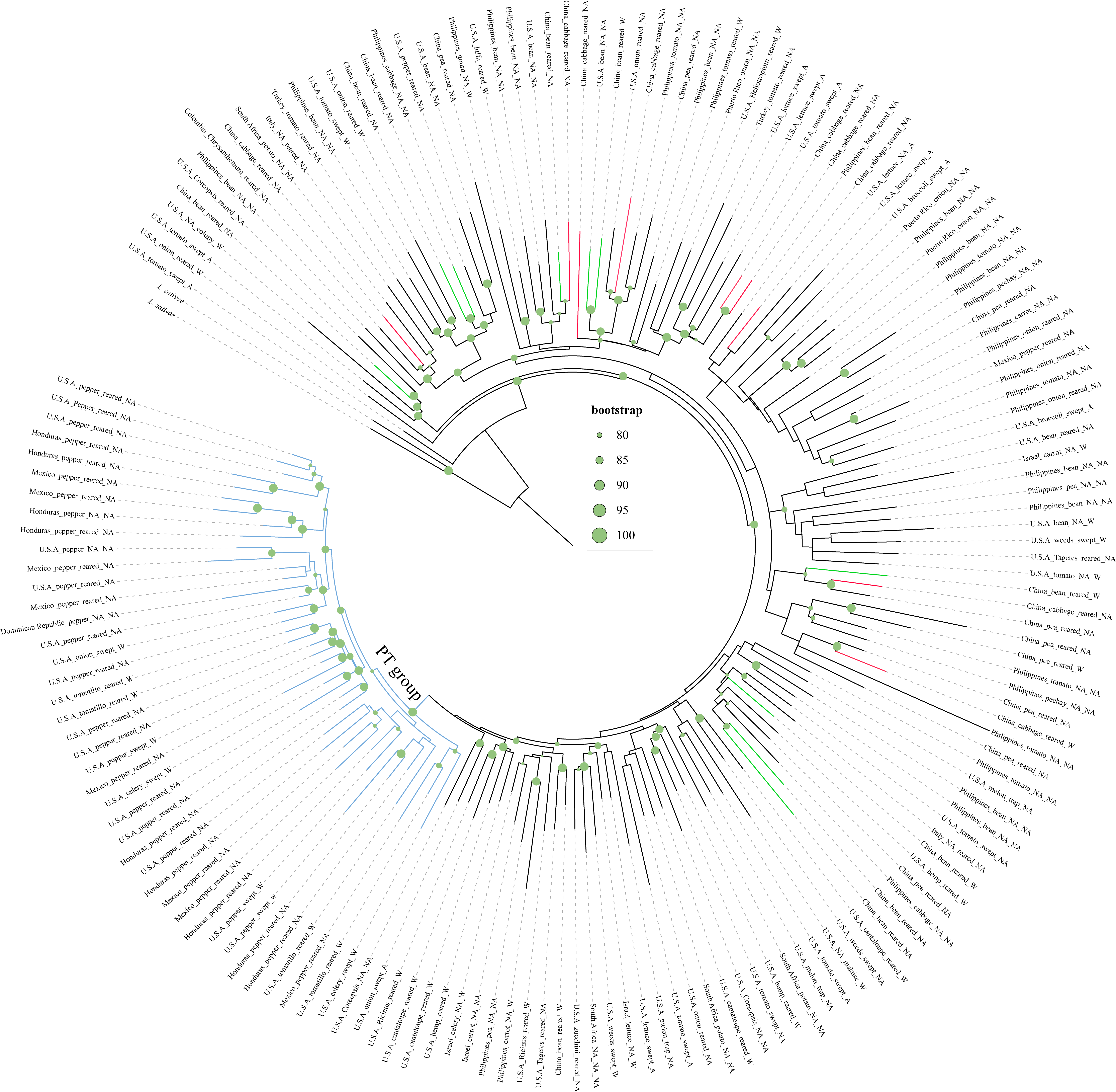
**

**Figure S1** Maximum likelihood tree within 169 *L. trifolii* individuals conducted in IQ-TREE. Tips on the tree represent geographic region, host plant, collection method, and mitochondrial clade. Ultrafast bootstrap value greater than 80% is marked by green circle on the node of phylogenetic tree. A blue clade represents monophyletic PT group including all the individuals from pepper and tomatillo, possible celery and onion. NA indicates not available for the corresponding information for the sample. The red branches show the individuals collected from cabbage plants in Jiangsu, China, while the green branches represent the individuals from bean hosts in Gansu, China.


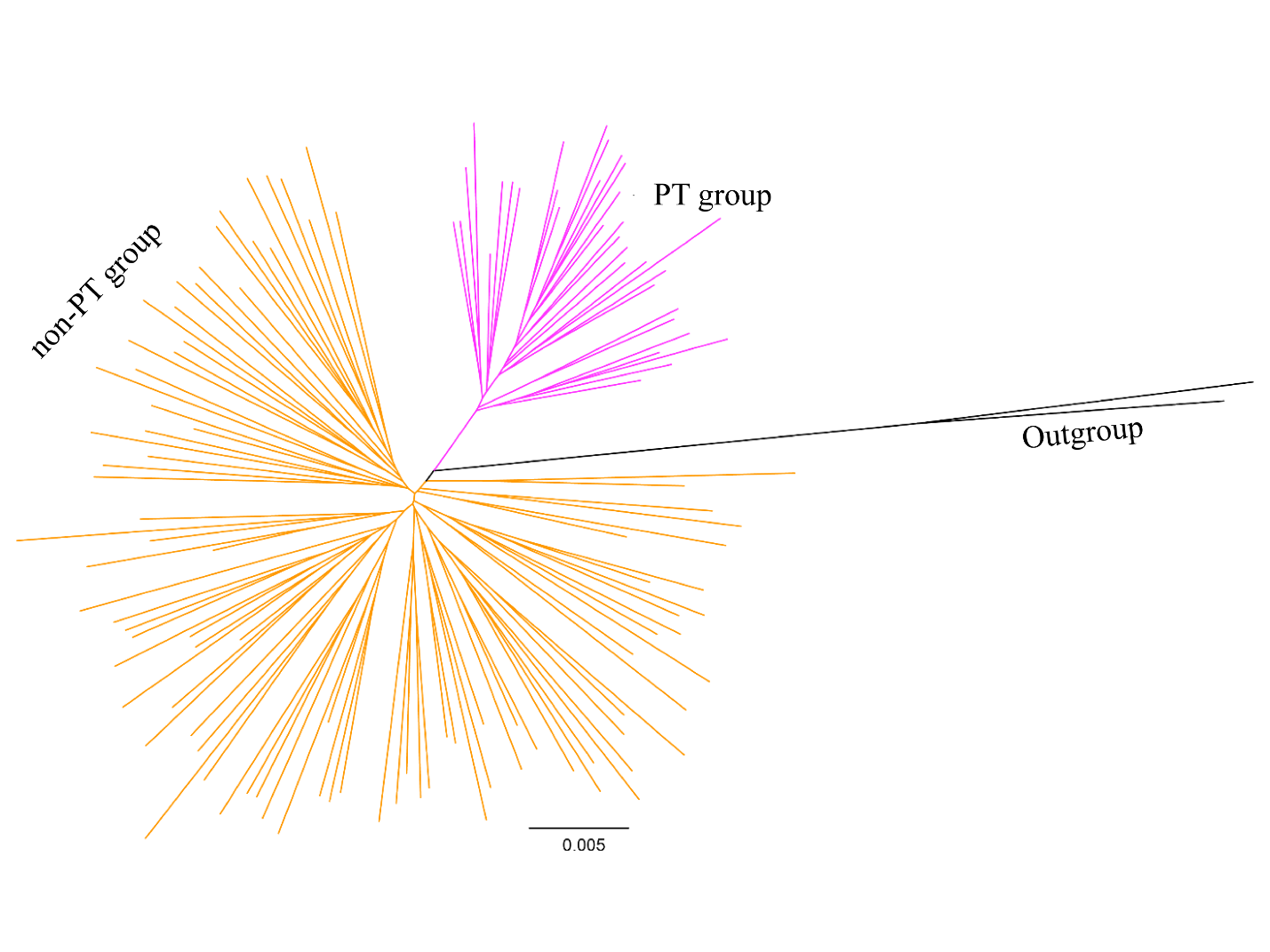


**Figure S2** Rooted neighbor-joining (NJ) tree for 128 samples implemented in SplitsTree4. Color pink and orange separately represent PT and non-PT group.


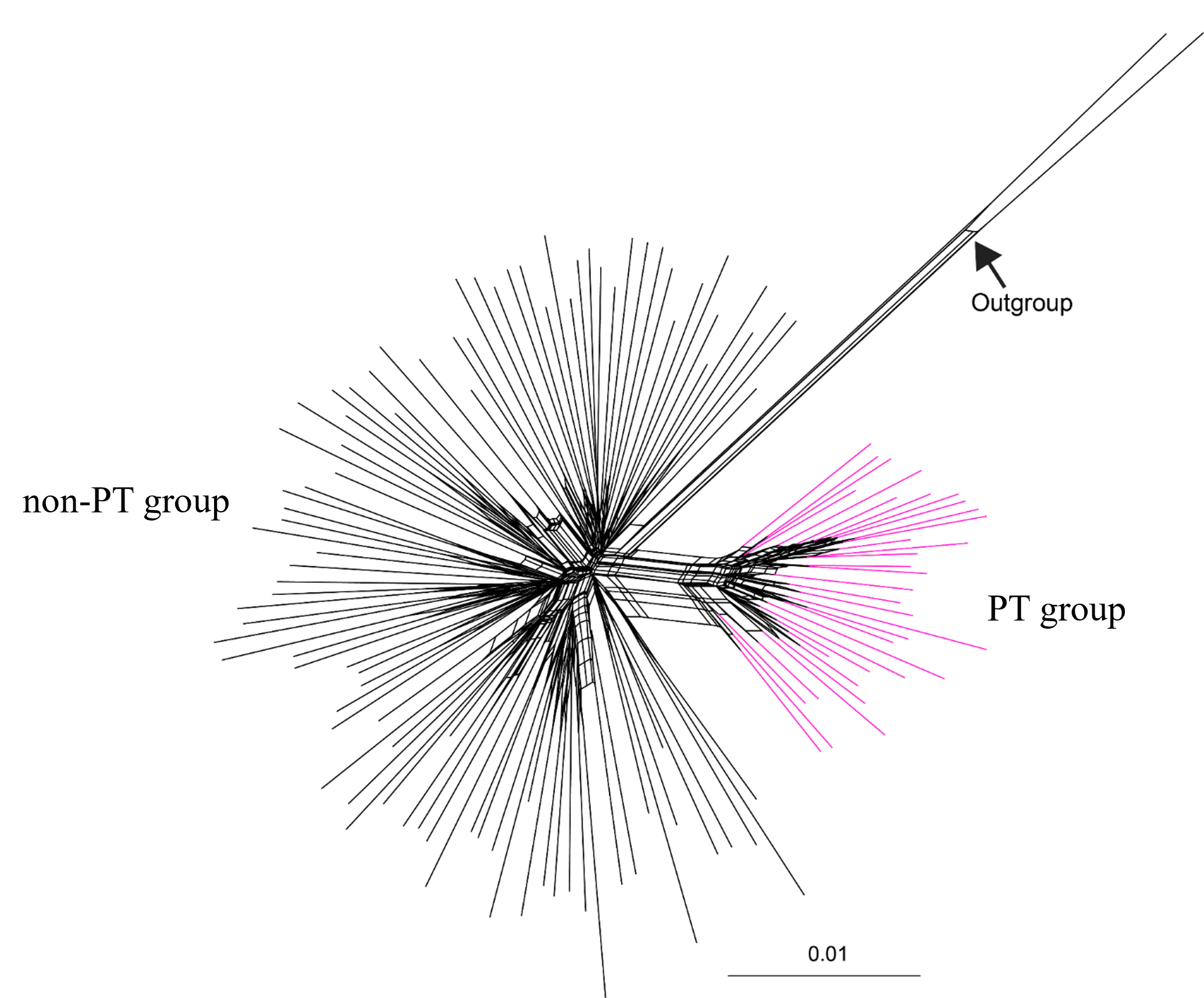


**Figure S3** Neighbor network of 128 individuals showing split conflicts with uncorrected-*p* distances inferred from program SplitsTree4.

**Table S1** Details of 171 samples covered in this study

| **Sample code** | **Species ID** | **Collection locality** | **Latitude, Longitude** | **Host plant** | **Plant family** | **Collection method** | **Life stage** | **Clade** |
| --- | --- | --- | --- | --- | --- | --- | --- | --- |
| AG0609 | *L. trifolii* | U.S.A: Maryland: Patuxent Research Refuge North Tract C-264 | 39.07, -76.77 | NA | NA | malaise | Adult | W |
| AG0634 | *L. trifolii* | U.S.A: Maryland: Aldis Clinton | 38.77, -76.88 | onion | Amaryllidaceae | reared | Adult | W |
| AG0635 | *L. trifolii* | U.S.A: Maryland: Aldis Clinton | 38.77, -76.88 | onion | Amaryllidaceae | reared | Adult | W |
| AG0673 | *L. trifolii* | Philippines | NA | bitter gourd | Cucurbitaceae | NA | Female | W |
| AG1453 | *L. trifolii* | U.S.A: California: San Luis Obispo Co. | 39.39, -120.76 | tomato | Solanaceae | swept | Adult | A |
| AG1483 | *L. trifolii* | U.S.A: California: Ventura | 34.76, -119.37 | tomato | Solanaceae | swept | Adult | A |
| AG1487 | *L. trifolii* | U.S.A: California: Ventura | 34.76, -119.37 | tomato | Solanaceae | swept | Adult | A |
| AG1489 | *L. trifolii* | U.S.A: California: Ventura | 34.76, -119.37 | broccoli | Brassicaceae | swept | Adult | A |
| AG1491 | *L. trifolii* | U.S.A: California: Ventura | 34.76, -119.37 | celery | Apiaceae | swept | Adult | W |
| AG1492 | *L. trifolii* | U.S.A: California: Ventura | 34.76, -119.37 | celery | Apiaceae | swept | Adult | W |
| AG1498 | *L. trifolii* | U.S.A: California: Ventura | 34.76, -119.37 | onion | Amaryllidaceae | swept | Adult | A |
| AG1500 | *L. trifolii* | U.S.A: California: Ventura | 34.76, -119.37 | onion | Amaryllidaceae | swept | Adult | W |
| AG1502 | *L. trifolii* | U.S.A: California: Ventura | 34.76, -119.37 | pepper | Solanaceae | swept | Adult | W-P |
| AG1503 | *L. trifolii* | U.S.A: California: Ventura | 34.76, -119.37 | pepper | Solanaceae | swept | Adult | W-P |
| AG1504 | *L. trifolii* | U.S.A: California: Ventura | 34.76, -119.37 | pepper | Solanaceae | swept | Adult | W-P |
| AG1506 | *L. trifolii* | U.S.A: California: Ventura | 34.76, -119.37 | tomato | Solanaceae | swept | Adult | A |
| AG1510 | *L. trifolii* | U.S.A: California: Fresno | 38.50, -120.25 | lettuce | Asteraceae | swept | Adult | A |
| AG1513 | *L. trifolii* | U.S.A: California: Fresno | 38.50, -120.25 | lettuce | Asteraceae | NA | NA | A |
| AG1518 | *L. trifolii* | U.S.A: California: Fresno | 38.50, -120.25 | broccoli | Brassicaceae | swept | Adult | A |
| AG1529 | *L. trifolii* | U.S.A: California: Fresno | 38.50, -120.25 | lettuce | Asteraceae | swept | Adult | A |
| AG1532 | *L. trifolii* | U.S.A: California: Fresno | 38.50, -120.25 | lettuce | Asteraceae | swept | Adult | A |
| AG1540 | *L. trifolii* | U.S.A: California: Fresno | 38.50, -120.25 | tomato | Solanaceae | swept | Adult | A |
| AG1551 | *L. trifolii* | U.S.A: California: Monterey | 36.83, -121.74 | lettuce | Asteraceae | swept | Adult | A |
| AG1553 | *L. trifolii* | U.S.A: Florida: Immokalee | 26.60, -81.46 | tomato | Solanaceae | swept | Adult | W |
| AG1554 | *L. trifolii* | U.S.A: Florida: Homestead: Miami-Dade Co. | 25.66, -80.53 | bean | Fabaceae | NA | NA | W |
| AG1560 | *L. trifolii* | U.S.A: Florida: Immokalee | 26.60, -81.46 | weeds | NA | swept | Adult | W |
| AG1562 | *L. trifolii* | U.S.A: Florida: Immokalee | 26.60, -81.46 | weeds | NA | swept | Adult | W |
| AG1565 | *L. trifolii* | U.S.A: Florida: Bradenton | 27.83, -82.58 | tomato | Solanaceae | NA | NA | W |
| AG1586 | *L. trifolii* | U.S.A: Florida: Orange Co.: Lake Buena Vista | 28.35, -81.48 | marigold | Asteraceae | reared | Female | W |
| AG1587 | *L. trifolii* | U.S.A: Florida: Orange County: Lake Buena Vista | 28.35, -81.48 | marigold | Asteraceae | reared | Female | W |
| AG1631 | *L. trifolii* | U.S.A: Florida: Immokalee | 26.60, -81.46 | pepper | Solanaceae | reared | Adult | W-P |
| AG2172 | *L. trifolii* | Israel: Kibbutz | 32.42, 34.84 | carrot | Apiaceae | NA | NA | W |
| FF2000 | *L. trifolii* | U.S.A: Florida: Epcot | 28.37, -81.54 | celery | NA | NA | NA | W |
| FF7100 | *L. trifolii* | Israel: Gilat | 31.32, 34.65 | celery | Apiaceae | NA | NA | W |
| FF9000 | *L. trifolii* | Israel: Gilat | 31.32, 34.65 | lettuce | Asteraceae | NA | NA | W |
| GSLtr1 | *L. trifolii* | China: Gansu: Shuichuan Co. | 37.14, 104.33 | bean | Fabaceae | reared | Male | NA |
| GSLtr10 | *L. trifolii* | China: Gansu: Shuichuan Co. | 37.14, 104.33 | bean | Fabaceae | reared | Female | NA |
| GSLtr2 | *L. trifolii* | China: Gansu: Shuichuan Co. | 37.14, 104.33 | bean | Fabaceae | reared | Male | NA |
| GSLtr3 | *L. trifolii* | China: Gansu: Shuichuan Co. | 37.14, 104.33 | bean | Fabaceae | reared | Male | NA |
| GSLtr4 | *L. trifolii* | China: Gansu: Shuichuan Co. | 37.14, 104.33 | bean | Fabaceae | reared | Male | NA |
| GSLtr5 | *L. trifolii* | China: Gansu: Shuichuan Co. | 37.14, 104.33 | bean | Fabaceae | reared | Male | NA |
| GSLtr6 | *L. trifolii* | China: Gansu: Shuichuan Co. | 37.14, 104.33 | bean | Fabaceae | reared | Female | W |
| GSLtr7 | *L. trifolii* | China: Gansu: Shuichuan Co. | 37.14, 104.33 | bean | Fabaceae | reared | Female | W |
| GSLtr8 | *L. trifolii* | China: Gansu: Shuichuan Co. | 37.14, 104.33 | bean | Fabaceae | reared | Female | W |
| GSLtr9 | *L. trifolii* | China: Gansu: Shuichuan Co. | 37.14, 104.33 | bean | Fabaceae | reared | Female | W |
| HNLtr1 | *L. trifolii* | China: Hainan: Bailian Co. | 19.51, 109.57 | pea | Fabaceae | reared | Male | NA |
| HNLtr10 | *L. trifolii* | China: Hainan: Bailian Co. | 19.51, 109.57 | pea | Fabaceae | reared | Female | NA |
| HNLtr3 | *L. trifolii* | China: Hainan: Bailian Co. | 19.51, 109.57 | pea | Fabaceae | reared | Male | W |
| HNLtr4 | *L. trifolii* | China: Hainan: Bailian Co. | 19.51, 109.57 | pea | Fabaceae | reared | Male | NA |
| HNLtr5 | *L. trifolii* | China: Hainan: Bailian Co. | 19.51, 109.57 | pea | Fabaceae | reared | Male | NA |
| HNLtr6 | *L. trifolii* | China: Hainan: Bailian Co. | 19.51, 109.57 | pea | Fabaceae | reared | Female | NA |
| HNLtr7 | *L. trifolii* | China: Hainan: Bailian Co. | 19.51, 109.57 | pea | Fabaceae | reared | Female | NA |
| HNLtr8 | *L. trifolii* | China: Hainan: Bailian Co. | 19.51, 109.57 | pea | Fabaceae | reared | Female | NA |
| HNLtr9 | *L. trifolii* | China: Hainan: Bailian Co. | 19.51, 109.57 | pea | Fabaceae | reared | Female | NA |
| HSC28 | *L. trifolii* | Turkey: Antalya: Hacıaliler | 36.93, 30.84 | tomato | Solanaceae | reared | Adult | NA |
| HSC29 | *L. trifolii* | Turkey: Antalya: Hacıaliler | 36.93, 30.84 | tomato | Solanaceae | reared | Adult | NA |
| JL15s | *L. trifolii* | U.S.A: North Carolina: Beaufort | 34.71, -76.66 | hemp | Cannabaceae | reared | Adult | W |
| JL17 | *L. trifolii* | U.S.A: University of Georgia | 33.94, -83.37 | heliotrope | Boraginaceae | reared | Adult | W |
| JL18 | *L. trifolii* | U.S.A: University of Georgia | 33.94, -83.37 | castor bean | Euphorbiaceae | reared | Adult | W |
| JL19 | *L. trifolii* | U.S.A: North Carolina: Raleigh | 35.89, -78.71 | luffa | Cucurbitaceae | reared | Adult | W |
| JL23 | *L. trifolii* | U.S.A: North Carolina: Wayne Co. | 35.44, -78.03 | cantaloupe | Cucurbitaceae | reared | Adult | W |
| JL24 | *L. trifolii* | U.S.A: North Carolina: Wayne Co. | 35.44, -78.03 | cantaloupe | Cucurbitaceae | reared | Adult | W |
| JL25 | *L. trifolii* | U.S.A: California: University of California Davis West Village: Hutchison Pl, Davis | 38.53, -121.77 | tomatillo | Solanaceae | reared | Male | W-P |
| JL26 | *L. trifolii* | U.S.A: California: University of California Davis West Village: Hutchison Pl, Davis | 38.53, -121.77 | tomatillo | Solanaceae | reared | Male | W-P |
| JL28 | *L. trifolii* | U.S.A: California: University of California Davis West Village: Hutchison Pl, Davis | 38.53, -121.77 | tomatillo | Solanaceae | reared | Male | W-P |
| JL29 | *L. trifolii* | U.S.A: California: University of California Davis West Village: Hutchison Pl, Davis | 38.53, -121.77 | tomatillo | Solanaceae | reared | Male | W-P |
| JL31 | *L. trifolii* | U.S.A: North Carolina: Beaufort: Belhaven: 3264 Beech Ridge Rd, C.Rouse | 35.57, -76.58 | hemp | Cannabaceae | reared | Adult | W |
| JL32 | *L. trifolii* | U.S.A: North Carolina: Beaufort: Belhaven: 3264 Beech Ridge Rd, C.Rouse | 35.57, -76.58 | hemp | Cannabaceae | reared | Male | W |
| JL33 | *L. trifolii* | U.S.A: Georgia: University of Georgia | 33.94, -83.37 | castor bean | Euphorbiaceae | reared | Adult | W |
| JL34 | *L. trifolii* | U.S.A: North Carolina: Seven Springs | 35.26, -77.84 | cantaloupe | Cucurbitaceae | reared | Adult | W |
| JL35 | *L. trifolii* | U.S.A: North Carolina: Seven Springs | 35.26, -77.84 | cantaloupe | Cucurbitaceae | reared | Adult | W |
| JSLtr10 | *L. trifolii* | China: Jiangsu: Zhenjiang City | 32.18, 119.60 | cabbage | Brassicaceae | reared | Female | NA |
| JSLtr2 | *L. trifolii* | China: Jiangsu: Zhenjiang City | 32.18, 119.60 | cabbage | Brassicaceae | reared | Male | NA |
| JSLtr3 | *L. trifolii* | China: Jiangsu: Zhenjiang City | 32.18, 119.60 | cabbage | Brassicaceae | reared | Male | NA |
| JSLtr4 | *L. trifolii* | China: Jiangsu: Zhenjiang City | 32.18, 119.60 | cabbage | Brassicaceae | reared | Male | NA |
| JSLtr5 | *L. trifolii* | China: Jiangsu: Zhenjiang City | 32.18, 119.60 | cabbage | Brassicaceae | reared | Male | NA |
| JSLtr6 | *L. trifolii* | China: Jiangsu: Zhenjiang City | 32.18, 119.60 | cabbage | Brassicaceae | reared | Female | NA |
| JSLtr7 | *L. trifolii* | China: Jiangsu: Zhenjiang City | 32.18, 119.60 | cabbage | Brassicaceae | reared | Female | NA |
| JSLtr8 | *L. trifolii* | China: Jiangsu: Zhenjiang City | 32.18, 119.60 | cabbage | Brassicaceae | reared | Female | NA |
| JSLtr9 | *L. trifolii* | China: Jiangsu: Zhenjiang City | 32.18, 119.60 | cabbage | Brassicaceae | reared | Female | W |
| LFL2 | *L. trifolii* | U.S.A: Florida: Alachua Co.: Farmer Brown's field Rt. 301, Orange Heights | 29.71, -82.13 | zucchini | Cucurbitaceae | reared | Larva | W |
| LFL7 | *L. trifolii* | U.S.A: Florida: Immokalee | 26.60, -81.46 | pepper | Solanaceae | reared | Larva | W-P |
| LFL9 | *L. trifolii* | U.S.A: Florida: Homestead | 25.46, -80.41 | bean | Fabaceae | reared | Larva | W |
| Lmx3 | *L. trifolii* | Mexico: Tamaulipas St.: Tampico Valley | 22.52, -97.78 | pepper | Solanaceae | reared | Adult | W-P |
| Lmx4 | *L. trifolii* | Mexico: Tamaulipas St.: Tampico Valley | 22.52, -97.78 | pepper | Solanaceae | reared | Adult | W-P |
| LNY2 | *L. trifolii* | U.S.A: New York: Elba Muck Bays bag | 40.74, -73.92 | onion | Amaryllidaceae | reared | Larva | A |
| LNY6 | *L. trifolii* | U.S.A: New York: Newark | 41.20, -74.21 | onion | Amaryllidaceae | reared | Larva | A |
| LPPQ67 | *L. trifolii* | Colombia | 1.71, -73.19 | chrysanthemum | Asteraceae | reared | Larva | W |
| LPPQ71 | *L. trifolii* | Dominican Republic | 20.29, -69.90 | pepper | Solanaceae | NA | Pupa | W-P |
| Lsa15 | *L. trifolii* | Honduras: El Zamorano | 14.31, -86.95 | pepper | Solanaceae | reared | Larva | W-P |
| Lsa16 | *L. trifolii* | Honduras: El Zamorano | 14.31, -86.95 | pepper | Solanaceae | reared | Larva | W-P |
| Ltr105 | *L. trifolii* | South Africa: Western Cape Pro.: Lamberts Bay | -31.97, 18.33 | potato | Solanaceae | NA | NA | W |
| Ltr11 | *L. trifolii* | South Africa | -28.27, 24.14 | NA | NA | NA | NA | W |
| Ltr112 | *L. trifolii* | U.S.A: Florida: Immokalee | 26.60, -81.46 | pepper | Solanaceae | reared | Larva | W-P |
| Ltr113 | *L. trifolii* | U.S.A: Florida: Immokalee | 26.60, -81.46 | pepper | Solanaceae | reared | Larva | W-P |
| Ltr114 | *L. trifolii* | U.S.A: Florida: Immokalee | 26.60, -81.46 | pepper | Solanaceae | reared | Larva | W-P |
| Ltr115 | *L. trifolii* | U.S.A: Florida: Immokalee | 26.60, -81.46 | pepper | Solanaceae | reared | Larva | W-P |
| Ltr116 | *L. trifolii* | U.S.A: Florida: Immokalee | 26.60, -81.46 | pepper | Solanaceae | reared | Larva | W-P |
| Ltr117 | *L. trifolii* | U.S.A: Florida: Immokalee | 26.60, -81.46 | pepper | Solanaceae | reared | Larva | W-P |
| Ltr118 | *L. trifolii* | U.S.A: Florida: Immokalee | 26.60, -81.46 | pepper | Solanaceae | reared | Larva | W-P |
| Ltr119 | *L. trifolii* | U.S.A: Florida: Immokalee | 26.60, -81.46 | pepper | Solanaceae | reared | Larva | W-P |
| Ltr12 | *L. trifolii* | Israel | NA | carrot | Apiaceae | NA | NA | W-P |
| Ltr121 | *L. trifolii* | Mexico: Tamaulipas: Tampico Valley | 25.86, -98.97 | pepper | Solanaceae | reared | Pupa | W |
| Ltr122 | *L. trifolii* | Mexico: Tamaulipas: Tampico Valley | 25.86, -98.97 | pepper | Solanaceae | reared | Pupa | W-P |
| Ltr123 | *L. trifolii* | Mexico: Tamaulipas: Tampico Valley | 25.86, -98.97 | pepper | Solanaceae | reared | Pupa | W-P |
| Ltr124 | *L. trifolii* | Mexico: Tamaulipas: Tampico Valley | 25.86, -98.97 | pepper | Solanaceae | reared | Pupa | W-P |
| Ltr125 | *L. trifolii* | Mexico: Tamaulipas: Tampico Valley | 25.86, -98.97 | pepper | Solanaceae | reared | Pupa | W-P |
| Ltr126 | *L. trifolii* | Mexico: Tamaulipas: Tampico Valley | 25.86, -98.97 | pepper | Solanaceae | reared | Pupa | W-P |
| Ltr127 | *L. trifolii* | Mexico: Tamaulipas: Tampico Valley | 25.86, -98.97 | pepper | Solanaceae | reared | Pupa | W-P |
| Ltr128 | *L. trifolii* | Honduras: El Zamorano | 14.00, -86.99 | pepper | Solanaceae | reared | Larva | W-P |
| Ltr129 | *L. trifolii* | Honduras: El Zamorano | 14.00, -86.99 | pepper | Solanaceae | reared | Larva | W-P |
| Ltr13 | *L. trifolii* | U.S.A: California: Davis | 38.54, -121.73 | bean | Fabaceae | NA | NA | A |
| Ltr130 | *L. trifolii* | Honduras: El Zamorano | 14.00, -86.99 | pepper | Solanaceae | reared | larva | W-P |
| Ltr131 | *L. trifolii* | Honduras: El Zamorano | 14.00, -86.99 | pepper | Solanaceae | reared | larva | W-P |
| Ltr132 | *L. trifolii* | Honduras: El Zamorano | 14.00, -86.99 | pepper | Solanaceae | NA | larva | W-P |
| Ltr15 | *L. trifolii* | U.S.A: California: Trumble colony, north pop | 37.81, -121.21 | pepper | Solanaceae | reared | Adult | W-P |
| Ltr17 | *L. trifolii* | Italy | NA | NA | NA | reared | Adult | W |
| Ltr18 | *L. trifolii* | Italy | NA | NA | NA | reared | Adult | W |
| Ltr20 | *L. trifolii* | Puerto Rico | NA | onion | Amaryllidaceae | NA | NA | W |
| Ltr22 | *L. trifolii* | U.S.A: Arizona: Yuma | 32.66, -114.51 | melon | Cucurbitaceae | trap | Adult | A |
| Ltr27 | *L. trifolii* | South Africa: Western Cape Pro.: Lamberts Bay | -32.09, 18.32 | potato | Solanaceae | NA | NA | W |
| Ltr28 | *L. trifolii* | Honduras: El Zamorano | 14.00, -86.98 | pepper | Solanaceae | reared | Larva | W-P |
| Ltr29 | *L. trifolii* | Honduras: El Zamorano | 14.00, -86.98 | pepper | Solanaceae | reared | larva | W-P |
| Ltr30 | *L. trifolii* | U.S.A: North Carolina | 35.097, -78.90 | tickseed | Asteraceae | reared | Adult | W |
| Ltr34 | *L. trifolii* | U.S.A: Florida: Immokalee | 26.60, -81.46 | pepper | Solanaceae | NA | NA | W-P |
| Ltr35 | *L. trifolii* | U.S.A: Florida: Immokalee | 26.60, -81.46 | tomato | Solanaceae | swept | Adult | W |
| Ltr36 | *L. trifolii* | U.S.A: Florida: Immokalee | 26.60, -81.46 | weeds | NA | swept | Adult | W |
| Ltr37 | *L. trifolii* | U.S.A: Florida | 28.93, -81.60 | tomato | Solanaceae | swept | Adult | W |
| Ltr44 | *L. trifolii* | U.S.A: California: Trumble colony: north pop | 37.81, -121.21 | pepper | Solanaceae | reared | Adult | NA |
| Ltr50 | *L. trifolii* | U.S.A: California: Davis | 38.54, -121.73 | bean | Fabaceae | NA | NA | A |
| Ltr51 | *L. trifolii* | Puerto Rico | NA | onion | Amaryllidaceae | NA | NA | W |
| Ltr52 | *L. trifolii* | Puerto Rico | NA | onion | Amaryllidaceae | NA | NA | W |
| Ltr55 | *L. trifolii* | South Africa: Western Cape Pro.: Lamberts Bay | -31.97, 18.33 | potato | Solanaceae | NA | NA | W |
| Ltr57 | *L. trifolii* | Philippines: Nueva Ecija Pro.:San Jose City | 15.82, 121.00 | onion | Amaryllidaceae | reared | Adult | W |
| Ltr58 | *L. trifolii* | Philippines: Nueva Ecija Pro.:San Jose City | 15.82, 121.00 | onion | Amaryllidaceae | reared | Adult | W |
| Ltr59 | *L. trifolii* | Philippines: Nueva Ecija Pro.:San Jose City | 15.82, 121.00 | onion | Amaryllidaceae | reared | Adult | W |
| Ltr62 | *L. trifolii* | U.S.A: Arizona: Yuma | 32.66, -114.51 | melon | Cucurbitaceae | trap | Adult | A |
| Ltr63 | *L. trifolii* | U.S.A: Arizona: Yuma | 32.66, -114.51 | melon | Cucurbitaceae | trap | Adult | NA |
| Ltr64 | *L. trifolii* | U.S.A: North Carolina | 35.097, -78.90 | tickseed | Asteraceae | NA | NA | W |
| Ltr65 | *L. trifolii* | U.S.A: North Carolina | 35.097, -78.90 | coreopsis | Asteraceae | NA | NA | W |
| Ltr69 | *L. trifolii* | U.S.A: California: Davis | 38.54, -121.73 | bean | Fabaceae | NA | NA | A |
| PH1080 | *L. trifolii* | Philippines:Pangasinan pro: Urdaneta City: cabuloan | 15.99, 120.57 | tomato | Solanaceae | reared | NA | W |
| PH1230 | *L. trifolii* | Philippines: Parañaque City: Thomas street | 14.48, 121.00 | bean | Fabaceae | reared | NA | W |
| PH1580 | *L. trifolii* | Philippines: Nueva | 20.50, 121.91 | carrot | Apiaceae | NA | NA | W |
| PH5400 | *L. trifolii* | Philippines | NA | tomato | Solanaceae | NA | NA | W |
| RJ119 | *L. trifolii* | Philippines: Nueva Ecija Pro.:San Jose City | 15.82, 121.00 | bean | Fabaceae | NA | NA | W |
| RJ120 | *L. trifolii* | Philippines: Nueva Ecija Pro.:San Jose City | 15.82, 121.00 | bean | Fabaceae | NA | NA | W |
| RJ142 | *L. trifolii* | Philippines: Ifugao Pro. | 19.07, 121.24 | pechay | Brassicaceae | NA | NA | W |
| RJ143 | *L. trifolii* | Philippines: Ifugao Pro. | 19.07, 121.24 | pechay | Brassicaceae | NA | NA | W |
| RJ146 | *L. trifolii* | Philippines: Ifugao Pro. | 19.07, 121.24 | bean | Fabaceae | NA | NA | W |
| RJ147 | *L. trifolii* | Philippines: Ifugao Pro. | 19.07, 121.24 | bean | Fabaceae | NA | NA | W |
| RJ192 | *L. trifolii* | Philippines: Nueva Vizcaya Pro. | 20.92, 120.62 | bean | Fabaceae | NA | NA | W |
| RJ193 | *L. trifolii* | Philippines: Nueva Vizcaya Pro. | 20.92, 120.62 | bean | Fabaceae | NA | NA | W |
| RJ203 | *L. trifolii* | Philippines: Pangainan Pro. | 16.03, 120.23 | bean | Fabaceae | NA | NA | W |
| RJ205 | *L. trifolii* | Philippines: Pangainan Pro. | 16.03, 120.23 | bean | Fabaceae | NA | NA | W |
| RJ218 | *L. trifolii* | Philippines: Mountain Pro. | 14.55, 121.05 | tomato | Solanaceae | NA | NA | W |
| RJ219 | *L. trifolii* | Philippines: Mountain Pro. | 14.55, 121.05 | tomato | Solanaceae | NA | NA | W |
| RJ25 | *L. trifolii* | Philippines: Benguet Pro. | 24.02, 122.44 | bean | Fabaceae | NA | NA | W |
| RJ280 | *L. trifolii* | Philippines: Nueva Ecija Pro. | 18.96, 121.33 | cabbage | Brassicaceae | NA | NA | W |
| RJ281 | *L. trifolii* | Philippines: Nueva Ecija Pro. | 18.96, 121.33 | cabbage | Brassicaceae | NA | NA | W |
| RJ5 | *L. trifolii* | Philippines: Abra Pro. | 19.58, 121.03 | tomato | Solanaceae | NA | NA | W |
| RJ53 | *L. trifolii* | Philippines: Nueva Ecija Pro. | 18.96, 121.33 | bean | Fabaceae | NA | NA | W |
| RJ61 | *L. trifolii* | Philippines: Nueva Ecija Pro. | 18.96, 121.33 | carrot | Apiaceae | NA | NA | W |
| RJ63 | *L. trifolii* | Philippines: Nueva Ecija Pro. | 18.96, 121.33 | bean | Fabaceae | NA | NA | W |
| RJ64 | *L. trifolii* | Philippines: Nueva Ecija Pro. | 18.96, 121.33 | bean | Fabaceae | NA | NA | W |
| RJ73 | *L. trifolii* | Philippines: Benguet Pro. | 24.02, 122.44 | tomato | Solanaceae | NA | NA | W |
| RJ74 | *L. trifolii* | Philippines: Benguet Pro. | 24.02, 122.44 | tomato | Solanaceae | NA | NA | W |
| RJ89 | *L. trifolii* | Philippines: Benguet Pro. | 24.02, 122.44 | pea | Fabaceae | NA | NA | W |
| RJ90 | *L. trifolii* | Philippines: Benguet Pro. | 24.02, 122.44 | pea | Fabaceae | NA | NA | W |
| **AG1477** | *L. sativae* | U.S.A: California: Imperial | 33.12, -115.63 | lettuce | outgroup | NA | NA | sativae |
| **HSC26** | *L. sativae* | Turkey: Artvin: HS Civelek: Karalake Natural Park | 41.26, 41.71 | aquatic plants | outgroup | NA | NA | sativae |

**Notes:** sample IDs bolded are used as outgroups in this study. The meanings of abbreviations on the table above: Pro.: Province; Co.: County; NA: not available for the corresponding information.

**Table S2** Information of individuals from left to right on admixture plots in Figure S4

| **Order** | **Sample code** | **Collection region** | **Host plant** | **Collection method** | **No. of original SNPs** |
| --- | --- | --- | --- | --- | --- |
| 1 | LFL9 | U.S.A | bean | reared | 163871 |
| 2 | Ltr50 | U.S.A | bean | NA | 240490 |
| 3 | Ltr69 | U.S.A | bean | NA | 93556 |
| 4 | JL23 | U.S.A | cantaloupe | reared | 208192 |
| 5 | JL24 | U.S.A | cantaloupe | reared | 302576 |
| 6 | JL34 | U.S.A | cantaloupe | reared | 188476 |
| 7 | JL35 | U.S.A | cantaloupe | reared | 461059 |
| 8 | FF2000 | U.S.A | celery | colony | 114105 |
| 9 | Ltr30 | U.S.A | tickseed | reared | 229223 |
| 10 | Ltr64 | U.S.A | tickseed | NA | 356004 |
| 11 | Ltr65 | U.S.A | tickseed | NA | 196199 |
| 12 | JL15s | U.S.A | hemp | reared | 202215 |
| 13 | JL31 | U.S.A | hemp | reared | 212751 |
| 14 | JL32 | U.S.A | hemp | reared | 290434 |
| 15 | AG1551 | U.S.A | lettuce | swept | 263701 |
| 16 | Ltr22 | U.S.A | melon | trap | 268545 |
| 17 | Ltr62 | U.S.A | melon | trap | 113622 |
| 18 | Ltr63 | U.S.A | melon | trap | 291551 |
| 19 | AG0635 | U.S.A | onion | reared | 128024 |
| 20 | AG1498 | U.S.A | onion | swept | 188726 |
| 21 | AG1586 | U.S.A | marigold | reared | 168830 |
| 22 | AG1587 | U.S.A | marigold | reared | 313292 |
| 23 | AG1453 | U.S.A | tomato | swept | 225774 |
| 24 | AG1506 | U.S.A | tomato | swept | 125836 |
| 25 | Ltr35 | U.S.A | tomato | swept | 225331 |
| 26 | Ltr37 | U.S.A | tomato | swept | 251145 |
| 27 | AG1560 | U.S.A | weeds | swept | 205838 |
| 28 | AG1562 | U.S.A | weeds | swept | 178156 |
| 29 | Ltr36 | U.S.A | weeds | swept | 375060 |
| 30 | AG1491 | U.S.A | celery | swept | 100493 |
| 31 | AG1492 | U.S.A | celery | swept | 254318 |
| 32 | AG1500 | U.S.A | onion | swept | 306614 |
| 33 | JL25 | U.S.A | tomatillo | reared | 493339 |
| 34 | JL26 | U.S.A | tomatillo | reared | 490966 |
| 35 | JL28 | U.S.A | tomatillo | reared | 123903 |
| 36 | JL29 | U.S.A | tomatillo | reared | 237648 |
| 37 | AG1502 | U.S.A | pepper | swept | 213041 |
| 38 | AG1503 | U.S.A | pepper | swept | 104971 |
| 39 | AG1504 | U.S.A | pepper | swept | 253901 |
| 40 | AG1631 | U.S.A | pepper | reared | 221451 |
| 41 | LFL7 | U.S.A | pepper | reared | 230983 |
| 42 | Ltr112 | U.S.A | pepper | reared | 415536 |
| 43 | Ltr113 | U.S.A | pepper | reared | 473091 |
| 44 | Ltr114 | U.S.A | pepper | reared | 342879 |
| 45 | Ltr115 | U.S.A | pepper | reared | 382494 |
| 46 | Ltr116 | U.S.A | pepper | reared | 444204 |
| 47 | Ltr117 | U.S.A | pepper | reared | 341181 |
| 48 | Ltr118 | U.S.A | pepper | reared | 448141 |
| 49 | Ltr119 | U.S.A | pepper | reared | 391960 |
| 50 | Ltr34 | U.S.A | pepper | NA | 193068 |
| 51 | Ltr44 | U.S.A | pepper | colony | 253038 |
| 52 | Lsa15 | Honduras | pepper | reared | 194443 |
| 53 | Ltr128 | Honduras | pepper | reared | 323411 |
| 54 | Ltr129 | Honduras | pepper | reared | 319845 |
| 55 | Ltr130 | Honduras | pepper | reared | 415501 |
| 56 | Ltr131 | Honduras | pepper | reared | 379317 |
| 57 | Ltr132 | Honduras | pepper | NA | 283180 |
| 58 | Ltr28 | Honduras | pepper | reared | 173289 |
| 59 | Ltr29 | Honduras | pepper | reared | 335831 |
| 60 | Ltr122 | Mexico | pepper | reared | 215044 |
| 61 | Ltr125 | Mexico | pepper | reared | 187434 |
| 62 | Ltr126 | Mexico | pepper | reared | 169063 |
| 63 | Ltr127 | Mexico | pepper | reared | 318819 |
| 64 | Lmx3 | Mexico | pepper | reared | 97406 |
| 65 | Lmx4 | Mexico | pepper | reared | 93988 |
| 66 | Ltr20 | Puerto Rico | onion | NA | 106832 |
| 67 | Ltr52 | Puerto Rico | onion | NA | 245363 |
| 68 | GSLtr10 | China | bean | reared | 206092 |
| 69 | GSLtr2 | China | bean | reared | 94903 |
| 70 | GSLtr3 | China | bean | reared | 147166 |
| 71 | GSLtr4 | China | bean | reared | 203973 |
| 72 | GSLtr5 | China | bean | reared | 127937 |
| 73 | GSLtr6 | China | bean | reared | 213696 |
| 74 | GSLtr7 | China | bean | reared | 332800 |
| 75 | GSLtr8 | China | bean | reared | 161866 |
| 76 | GSLtr9 | China | bean | reared | 214741 |
| 77 | JSLtr10 | China | cabbage | reared | 149007 |
| 78 | JSLtr2 | China | cabbage | reared | 129424 |
| 79 | JSLtr4 | China | cabbage | reared | 172772 |
| 80 | JSLtr5 | China | cabbage | reared | 135139 |
| 81 | JSLtr7 | China | cabbage | reared | 232209 |
| 82 | JSLtr8 | China | cabbage | reared | 112423 |
| 83 | HNLtr10 | China | pea | reared | 143561 |
| 84 | HNLtr3 | China | pea | reared | 124207 |
| 85 | HNLtr4 | China | pea | reared | 146133 |
| 86 | HNLtr6 | China | pea | reared | 125099 |
| 87 | HNLtr8 | China | pea | reared | 155910 |
| 88 | PH1230 | Philippines | bean | reared | 117274 |
| 89 | RJ146 | Philippines | bean | NA | 91494 |
| 90 | RJ192 | Philippines | bean | NA | 326211 |
| 91 | RJ193 | Philippines | bean | NA | 294139 |
| 92 | RJ203 | Philippines | bean | NA | 215322 |
| 93 | RJ205 | Philippines | bean | NA | 159537 |
| 94 | RJ63 | Philippines | bean | NA | 155218 |
| 95 | RJ64 | Philippines | bean | NA | 225042 |
| 96 | RJ280 | Philippines | cabbage | NA | 165166 |
| 97 | RJ281 | Philippines | cabbage | NA | 125083 |
| 98 | RJ61 | Philippines | carrot | NA | 171148 |
| 99 | Ltr57 | Philippines | onion | reared | 92636 |
| 100 | Ltr58 | Philippines | onion | reared | 172504 |
| 101 | PH1080 | Philippines | tomato | reared | 134474 |
| 102 | RJ218 | Philippines | tomato | NA | 168453 |
| 103 | RJ219 | Philippines | tomato | NA | 143898 |
| 104 | RJ73 | Philippines | tomato | NA | 177624 |
| 105 | RJ74 | Philippines | tomato | NA | 116078 |
| 106 | Ltr105 | South Africa | potato | NA | 125365 |
| 107 | Ltr27 | South Africa | potato | NA | 236549 |
| 108 | Ltr55 | South Africa | potato | NA | 293359 |
| 109 | FF9000 | Israel | lettuce | NA | 300331 |

**Notes:** No. of SNPs is the original number of SNPs before filtering. Samples highlighted in the rows indicate the individuals from PT group in Figure 1.

**Table S3** Details of each locus over 400 bp in length for total 126 *L. trfolii* individuals

| **Loci name** | **Start position** | **End position** | **Sequence length (bp)** | **No. of individuals** | **No. of SNPs** |
| --- | --- | --- | --- | --- | --- |
| EOG76B94J | 183769 | 184233 | 464 | 8 | 6 |
| EOG71PCX2 | 232429 | 232926 | 497 | 6 | 10 |
| EOG7DG7ZS | 135307 | 135711 | 404 | 36 | 13 |
| EOG7TBPN4 | 21073 | 21567 | 494 | 14 | 15 |
| EOG7XQ8SP | 50599 | 51153 | 554 | 3 | 17 |
| EOG7SFVGX | 134734 | 135306 | 572 | 31 | 18 |
| EOG7QVXN9 | 266380 | 266907 | 527 | 115 | 19 |
| EOG7HBGWW | 178756 | 179439 | 683 | 8 | 20 |
| EOG7DK45Z | 161296 | 161937 | 641 | 25 | 20 |
| EOG7XHJ91 | 17851 | 18525 | 674 | 54 | 21 |
| EOG7135HJ | 81760 | 82206 | 446 | 5 | 23 |
| EOG796J2Z | 245389 | 246147 | 758 | 25 | 23 |
| EOG7S2CJZ | 26413 | 26847 | 434 | 46 | 25 |
| EOG75JB7R | 20425 | 21072 | 647 | 125 | 32 |
| EOG7WHTX4 | 223774 | 224229 | 455 | 17 | 33 |
| EOG7FJTMC | 150898 | 151404 | 506 | 64 | 35 |
| EOG7GND7M | 158545 | 158988 | 443 | 104 | 35 |
| EOG7PGRB4 | 266908 | 267396 | 488 | 69 | 38 |
| EOG7C06DB | 109207 | 109620 | 413 | 91 | 38 |
| EOG7TFJVZ | 9295 | 10065 | 770 | 17 | 39 |
| EOG71CS6J | 124018 | 124827 | 809 | 21 | 39 |
| EOG7GJJ0G | 236551 | 236961 | 410 | 79 | 39 |
| EOG70SJR2 | 101713 | 102648 | 935 | 11 | 40 |
| EOG7TV1W6 | 180625 | 181044 | 419 | 29 | 42 |
| EOG7X9ST5 | 33223 | 33744 | 521 | 36 | 42 |
| EOG76TNC4 | 122638 | 123069 | 431 | 68 | 42 |
| EOG7WB3F5 | 34144 | 34842 | 698 | 125 | 43 |
| EOG76XHKP | 45154 | 45726 | 572 | 29 | 44 |
| EOG72GBTR | 56239 | 56904 | 665 | 120 | 44 |
| EOG7R2P58 | 221731 | 222225 | 494 | 21 | 51 |
| EOG7VTR84 | 244267 | 244686 | 419 | 50 | 51 |
| EOG7CS59N | 261064 | 261492 | 428 | 64 | 52 |
| EOG72P39Z | 10066 | 10575 | 509 | 124 | 52 |
| EOG71098B | 137212 | 137697 | 485 | 42 | 53 |
| EOG70KT9J | 204763 | 205623 | 860 | 49 | 54 |
| EOG7135H1 | 25927 | 26412 | 485 | 112 | 55 |
| EOG7N99G7 | 95893 | 96546 | 653 | 26 | 56 |
| EOG7WX9W4 | 182191 | 182733 | 542 | 42 | 56 |
| EOG7161R6 | 214663 | 215088 | 425 | 36 | 57 |
| EOG7ND5Q6 | 58783 | 59199 | 416 | 59 | 57 |
| EOG79WMRT | 211363 | 211953 | 590 | 83 | 57 |
| EOG7DCCQF | 170920 | 171516 | 596 | 100 | 58 |
| EOG735FJ0 | 147736 | 148185 | 449 | 126 | 59 |
| EOG72VTSC | 120700 | 121158 | 458 | 50 | 60 |
| EOG7GV4Q1 | 54715 | 55530 | 815 | 85 | 60 |
| EOG7MM6SM | 177772 | 178248 | 476 | 52 | 61 |
| EOG7X164H | 11977 | 12729 | 752 | 40 | 62 |
| EOG71CS71 | 207751 | 208182 | 431 | 51 | 63 |
| EOG77HR1C | 72085 | 72693 | 608 | 126 | 64 |
| EOG71PCXT | 263089 | 263517 | 428 | 61 | 67 |
| EOG7KDRX0 | 237178 | 237921 | 743 | 35 | 68 |
| EOG7RRRTQ | 79708 | 80496 | 788 | 3 | 69 |
| EOG77B0JM | 84829 | 85470 | 641 | 21 | 69 |
| EOG7135H4 | 152326 | 152766 | 440 | 52 | 69 |
| EOG7RC8W5 | 252118 | 252618 | 500 | 12 | 70 |
| EOG7X6XKK | 92827 | 93408 | 581 | 111 | 70 |
| EOG7JTJGX | 103537 | 104268 | 731 | 61 | 73 |
| EOG75BKS1 | 66769 | 67248 | 479 | 37 | 74 |
| EOG7Q2ZRP | 246148 | 246648 | 500 | 126 | 74 |
| EOG73ZDDS | 170500 | 170919 | 419 | 58 | 75 |
| EOG7FZ9KB | 241603 | 242154 | 551 | 95 | 77 |
| EOG7VTR7X | 54304 | 54714 | 410 | 34 | 78 |
| EOG7W1GQM | 108769 | 109206 | 437 | 58 | 80 |
| EOG7SZ6PR | 59683 | 60270 | 587 | 56 | 81 |
| EOG748151 | 119641 | 120111 | 470 | 87 | 81 |
| EOG7ZDCF6 | 123436 | 124017 | 581 | 69 | 82 |
| EOG73C60C | 149302 | 149943 | 641 | 60 | 83 |
| EOG771CV3 | 202939 | 203370 | 431 | 51 | 84 |
| EOG7M10BP | 168928 | 169533 | 605 | 59 | 84 |
| EOG7NWHX0 | 142573 | 143049 | 476 | 76 | 84 |
| EOG7FVF90 | 258367 | 259119 | 752 | 97 | 84 |
| EOG79M124 | 1126 | 1593 | 467 | 56 | 85 |
| EOG74814Z | 95224 | 95892 | 668 | 64 | 85 |
| EOG7Z0WH3 | 219244 | 219651 | 407 | 48 | 86 |
| EOG7DG805 | 98959 | 99894 | 935 | 7 | 88 |
| EOG7Q8Q82 | 64792 | 65412 | 620 | 48 | 88 |
| EOG7D5N7Q | 32005 | 32646 | 641 | 90 | 88 |
| EOG7MDGBD | 178249 | 178755 | 506 | 54 | 89 |
| EOG7ZDCGS | 223363 | 223773 | 410 | 124 | 89 |
| EOG7R2P4T | 75862 | 76803 | 941 | 22 | 90 |
| EOG78136M | 251593 | 252117 | 524 | 20 | 93 |
| EOG700KW0 | 125998 | 126660 | 662 | 51 | 93 |
| EOG7R5JD1 | 193540 | 194022 | 482 | 52 | 93 |
| EOG7KQCN6 | 55531 | 56238 | 707 | 125 | 93 |
| EOG7RC8W4 | 130261 | 130887 | 626 | 46 | 94 |
| EOG7428NV | 67249 | 67791 | 542 | 65 | 94 |
| EOG7JF2JJ | 154804 | 155262 | 458 | 71 | 95 |
| EOG7T22XR | 16600 | 17145 | 545 | 45 | 96 |
| EOG7X163C | 212782 | 213312 | 530 | 69 | 96 |
| EOG7XHJ9R | 67792 | 68361 | 569 | 125 | 97 |
| EOG77X70F | 186388 | 186903 | 515 | 61 | 98 |
| EOG7MSZ85 | 15937 | 16599 | 662 | 36 | 99 |
| EOG793NTB | 39022 | 39966 | 944 | 49 | 100 |
| EOG70PPJ4 | 553 | 1125 | 572 | 115 | 100 |
| EOG764JPP | 48154 | 48741 | 587 | 70 | 101 |
| EOG7M9M25 | 217153 | 217902 | 749 | 126 | 101 |
| EOG7R5JCB | 247537 | 248277 | 740 | 27 | 102 |
| EOG7D2S16 | 149944 | 150525 | 581 | 75 | 102 |
| EOG7H4RDS | 63142 | 63804 | 662 | 44 | 103 |
| EOG7WTFN5 | 24550 | 25059 | 509 | 46 | 104 |
| EOG7K9WQ0 | 122017 | 122463 | 446 | 37 | 106 |
| EOG7GND77 | 257818 | 258366 | 548 | 93 | 106 |
| EOG7G25TJ | 177259 | 177771 | 512 | 105 | 108 |
| EOG78DK62 | 268144 | 268767 | 623 | 100 | 109 |
| EOG7KDRX6 | 146197 | 146877 | 680 | 43 | 110 |
| EOG71PCXP | 179674 | 180345 | 671 | 40 | 111 |
| EOG7FFZD6 | 166276 | 166866 | 590 | 70 | 111 |
| EOG7CKDTZ | 26848 | 27387 | 539 | 80 | 111 |
| EOG7G521J | 133540 | 134127 | 587 | 83 | 111 |
| EOG76B955 | 203734 | 204762 | 1028 | 126 | 111 |
| EOG73JXGG | 267397 | 268143 | 746 | 80 | 112 |
| EOG72ZQ0M | 172654 | 173232 | 578 | 93 | 112 |
| EOG7BWB5B | 138433 | 138876 | 443 | 68 | 114 |
| EOG7J4FT1 | 107335 | 108051 | 716 | 77 | 114 |
| EOG7KDRW9 | 104269 | 104874 | 605 | 119 | 114 |
| EOG7JTJHD | 233776 | 234213 | 437 | 27 | 115 |
| EOG7F2GDD | 94288 | 94857 | 569 | 84 | 115 |
| EOG7DG7ZX | 214093 | 214662 | 569 | 96 | 115 |
| EOG7JXDQM | 209869 | 210282 | 413 | 57 | 118 |
| EOG79WMRF | 185050 | 185544 | 494 | 120 | 118 |
| EOG76QS3P | 84208 | 84639 | 431 | 4 | 119 |
| EOG732K8G | 238177 | 238806 | 629 | 69 | 122 |
| EOG7ZH7Q2 | 158989 | 159441 | 452 | 124 | 125 |
| EOG7B3C6T | 38164 | 38778 | 614 | 98 | 127 |
| EOG7QS2FM | 151405 | 151833 | 428 | 124 | 129 |
| EOG7GBSHN | 92260 | 92826 | 566 | 42 | 132 |
| EOG74V7JJ | 87025 | 88299 | 1274 | 125 | 133 |
| EOG7Q03J0 | 205624 | 206214 | 590 | 71 | 134 |
| EOG7M9M1V | 169795 | 170499 | 704 | 60 | 135 |
| EOG7ZM3XQ | 243616 | 244266 | 650 | 124 | 135 |
| EOG7Z6MZP | 91732 | 92259 | 527 | 85 | 137 |
| EOG7RZH90 | 232927 | 233775 | 848 | 69 | 138 |
| EOG7T4Z69 | 157735 | 158265 | 530 | 106 | 138 |
| EOG7M9M2D | 155263 | 156000 | 737 | 66 | 139 |
| EOG7WHTX8 | 184234 | 184779 | 545 | 126 | 139 |
| EOG7SVBG6 | 175882 | 176310 | 428 | 26 | 141 |
| EOG7JF2J1 | 234214 | 234696 | 482 | 77 | 141 |
| EOG7Q8Q7X | 140794 | 141330 | 536 | 44 | 142 |
| EOG754V8D | 143338 | 143760 | 422 | 10 | 144 |
| EOG7XDP2P | 215089 | 215754 | 665 | 14 | 144 |
| EOG7KQCNM | 145678 | 146196 | 518 | 65 | 144 |
| EOG7G521S | 14188 | 14823 | 635 | 52 | 145 |
| EOG7DG7ZW | 8698 | 9294 | 596 | 59 | 145 |
| EOG73RNZ2 | 192661 | 193152 | 491 | 30 | 146 |
| EOG7D5N6Z | 254656 | 255363 | 707 | 22 | 148 |
| EOG72VTS8 | 46972 | 47886 | 914 | 119 | 148 |
| EOG75FG0M | 93409 | 94029 | 620 | 125 | 148 |
| EOG7MM6S0 | 77149 | 77856 | 707 | 60 | 149 |
| EOG75R2PZ | 5116 | 6249 | 1133 | 123 | 149 |
| EOG752025 | 165736 | 166275 | 539 | 120 | 150 |
| EOG73G26F | 90451 | 91731 | 1280 | 54 | 151 |
| EOG7ZH7PN | 141331 | 142188 | 857 | 85 | 152 |
| EOG7SZ6PV | 20017 | 20424 | 407 | 44 | 156 |
| EOG7QVXQ8 | 59200 | 59682 | 482 | 103 | 156 |
| EOG78WXCQ | 133003 | 133539 | 536 | 126 | 156 |
| EOG7J4FTQ | 148408 | 149301 | 893 | 71 | 157 |
| EOG7DG7Z7 | 86179 | 87024 | 845 | 90 | 158 |
| EOG7FNPT2 | 174013 | 174546 | 533 | 57 | 159 |
| EOG78DK5C | 80932 | 81387 | 455 | 13 | 163 |
| EOG735FH7 | 42499 | 43008 | 509 | 52 | 167 |
| EOG74RCBB | 131176 | 131901 | 725 | 69 | 167 |
| EOG7V4NJS | 175333 | 175881 | 548 | 75 | 167 |
| EOG7WMQ48 | 208183 | 208881 | 698 | 108 | 169 |
| EOG7096JQ | 134128 | 134733 | 605 | 48 | 170 |
| EOG7J4FT6 | 137698 | 138177 | 479 | 95 | 170 |
| EOG7NWHX2 | 188356 | 188889 | 533 | 43 | 171 |
| EOG754V90 | 182734 | 183435 | 701 | 67 | 171 |
| EOG72RZJ7 | 246934 | 247536 | 602 | 72 | 172 |
| EOG79PW85 | 222226 | 222843 | 617 | 100 | 172 |
| EOG7DZM52 | 32647 | 33222 | 575 | 91 | 173 |
| EOG7Q5V1N | 25441 | 25926 | 485 | 107 | 173 |
| EOG7QK9Z1 | 186904 | 187584 | 680 | 119 | 173 |
| EOG78HFDZ | 118336 | 118842 | 506 | 56 | 174 |
| EOG7FFZBV | 1594 | 2577 | 983 | 56 | 174 |
| EOG7DCCR4 | 62611 | 63141 | 530 | 63 | 174 |
| EOG7S57SQ | 21568 | 22005 | 437 | 67 | 174 |
| EOG7HQZWQ | 268768 | 269448 | 680 | 76 | 174 |
| EOG77B0JR | 85471 | 86178 | 707 | 91 | 175 |
| EOG7N0PQF | 206215 | 206988 | 773 | 59 | 176 |
| EOG70SJRP | 10864 | 11976 | 1112 | 126 | 177 |
| EOG7C06CB | 230395 | 231531 | 1136 | 76 | 178 |
| EOG76B94H | 257086 | 257538 | 452 | 15 | 180 |
| EOG72GBV3 | 8020 | 8697 | 677 | 54 | 181 |
| EOG7VTR7P | 80497 | 80931 | 434 | 55 | 182 |
| EOG7KHN5P | 36259 | 36966 | 707 | 94 | 182 |
| EOG73C5ZN | 138877 | 139683 | 806 | 102 | 183 |
| EOG7M3VKS | 35224 | 35643 | 419 | 54 | 188 |
| EOG7JB695 | 63805 | 64494 | 689 | 99 | 188 |
| EOG7P0D4K | 77857 | 78996 | 1139 | 126 | 188 |
| EOG7PW78W | 36967 | 37779 | 812 | 80 | 192 |
| EOG7TXX39 | 96547 | 97323 | 776 | 119 | 192 |
| EOG7PGR9F | 69547 | 70887 | 1340 | 7 | 193 |
| EOG786TP5 | 173233 | 174012 | 779 | 62 | 196 |
| EOG77B0HF | 227794 | 228864 | 1070 | 19 | 204 |
| EOG74V7K8 | 3184 | 3735 | 551 | 73 | 204 |
| EOG7TJF3D | 100258 | 100674 | 416 | 17 | 206 |
| EOG78136S | 113608 | 114828 | 1220 | 126 | 211 |
| EOG74FRM1 | 17146 | 17850 | 704 | 64 | 212 |
| EOG7MSZ7R | 111991 | 113298 | 1307 | 74 | 212 |
| EOG7X6XKB | 73771 | 74634 | 863 | 126 | 212 |
| EOG7V7HSX | 255364 | 256119 | 755 | 35 | 214 |
| EOG7K9WP5 | 153904 | 154803 | 899 | 58 | 214 |
| EOG7FRK2X | 98410 | 98958 | 548 | 85 | 215 |
| EOG7MSZ7Z | 206989 | 207750 | 761 | 24 | 217 |
| EOG7RNWKC | 118843 | 119640 | 797 | 30 | 217 |
| EOG7GJHZK | 125002 | 125997 | 995 | 23 | 219 |
| EOG7DZM4W | 3736 | 5115 | 1379 | 125 | 221 |
| EOG71KHNQ | 70888 | 71679 | 791 | 119 | 230 |
| EOG78WXBF | 53017 | 54069 | 1052 | 73 | 231 |
| EOG7PCW2B | 231532 | 232080 | 548 | 35 | 233 |
| EOG71KHPB | 191470 | 192342 | 872 | 81 | 234 |
| EOG7XQ8RK | 105991 | 107334 | 1343 | 107 | 235 |
| EOG7WMQ4Z | 211954 | 212781 | 827 | 123 | 239 |
| EOG7M10C1 | 229780 | 230394 | 614 | 83 | 244 |
| EOG7XQ8S8 | 40636 | 41340 | 704 | 92 | 244 |
| EOG75R2PW | 171517 | 172653 | 1136 | 123 | 244 |
| EOG754V82 | 185545 | 186387 | 842 | 43 | 246 |
| EOG793NTH | 209371 | 209868 | 497 | 114 | 246 |
| EOG748154 | 262642 | 263088 | 446 | 72 | 250 |
| EOG7Z9H69 | 135712 | 137211 | 1499 | 125 | 250 |
| EOG78137C | 261949 | 262641 | 692 | 86 | 257 |
| EOG73G262 | 6250 | 8019 | 1769 | 24 | 260 |
| EOG77TBR4 | 114829 | 115287 | 458 | 65 | 260 |
| EOG7D8HGG | 61138 | 62610 | 1472 | 126 | 261 |
| EOG703G2J | 88300 | 89268 | 968 | 119 | 263 |
| EOG7K71DV | 188890 | 190464 | 1574 | 125 | 270 |
| EOG7K71FM | 213313 | 214092 | 779 | 67 | 271 |
| EOG7B93PX | 146878 | 147735 | 857 | 68 | 272 |
| EOG75202B | 195505 | 196266 | 761 | 96 | 277 |
| EOG7VBD24 | 66106 | 66768 | 662 | 106 | 277 |
| EOG7RC8W1 | 19405 | 20016 | 611 | 62 | 278 |
| EOG7HJ7BV | 226153 | 227793 | 1640 | 125 | 283 |
| EOG7SRG6Q | 14824 | 15936 | 1112 | 23 | 284 |
| EOG7JHXRZ | 160477 | 161295 | 818 | 126 | 284 |
| EOG7WHTWC | 131902 | 133002 | 1100 | 61 | 287 |
| EOG700KVR | 108052 | 108768 | 716 | 37 | 289 |
| EOG7F5BN1 | 164530 | 165486 | 956 | 79 | 290 |
| EOG7R8DN3 | 126661 | 127260 | 599 | 105 | 296 |
| EOG786TPD | 263848 | 264549 | 701 | 78 | 300 |
| EOG7PW78R | 35644 | 36258 | 614 | 55 | 302 |
| EOG7HBGWS | 46393 | 46971 | 578 | 31 | 305 |
| EOG7RNWK0 | 100675 | 101712 | 1037 | 73 | 306 |
| EOG7Q03H3 | 45727 | 46392 | 665 | 90 | 307 |
| EOG7BKQDC | 208882 | 209370 | 488 | 45 | 312 |
| EOG7KT7W7 | 57466 | 58026 | 560 | 64 | 318 |
| EOG7M10BJ | 22006 | 22656 | 650 | 44 | 321 |
| EOG73C5ZF | 65413 | 66105 | 692 | 92 | 326 |
| EOG7BCZZ5 | 210283 | 210909 | 626 | 102 | 327 |
| EOG7VQVZX | 156316 | 157734 | 1418 | 125 | 333 |
| EOG72VTRX | 22657 | 23802 | 1145 | 121 | 336 |
| EOG7RK1B3 | 102649 | 103536 | 887 | 118 | 339 |
| EOG7W775H | 248617 | 249300 | 683 | 67 | 348 |
| EOG7R2P43 | 194023 | 195165 | 1142 | 116 | 348 |
| EOG7ZQ053 | 181045 | 182190 | 1145 | 81 | 352 |
| EOG7R2P4M | 187585 | 188355 | 770 | 77 | 354 |
| EOG7W4C0H | 253480 | 254292 | 812 | 77 | 354 |
| EOG789PXR | 259771 | 260745 | 974 | 121 | 356 |
| EOG7BCZZ2 | 159442 | 160200 | 758 | 17 | 357 |
| EOG7MM6RM | 264550 | 265080 | 530 | 44 | 361 |
| EOG7HFC4K | 121159 | 121803 | 644 | 51 | 365 |
| EOG74JMTJ | 196267 | 197358 | 1091 | 71 | 370 |
| EOG7B93PT | 176311 | 177258 | 947 | 118 | 376 |
| EOG74V7JF | 225025 | 226152 | 1127 | 27 | 381 |
| EOG74FRMM | 68848 | 69546 | 698 | 84 | 388 |
| EOG76XHK6 | 60271 | 60807 | 536 | 93 | 389 |
| EOG7VQVZV | 97324 | 98409 | 1085 | 44 | 391 |
| EOG77482B | 240883 | 241602 | 719 | 94 | 395 |
| EOG76J1MC | 18526 | 19404 | 878 | 23 | 396 |
| EOG7V1S9F | 109621 | 111075 | 1454 | 108 | 396 |
| EOG70WF15 | 39967 | 40635 | 668 | 42 | 397 |
| EOG7ZM3XD | 190465 | 191469 | 1004 | 91 | 412 |
| EOG7T22WW | 111319 | 111990 | 671 | 31 | 419 |
| EOG7135GS | 72694 | 73470 | 776 | 106 | 424 |
| EOG7B93PF | 234913 | 235854 | 941 | 97 | 427 |
| EOG7NH1X7 | 249301 | 250329 | 1028 | 126 | 427 |
| EOG7WQKCJ | 220849 | 221730 | 881 | 76 | 432 |
| EOG75201D | 23803 | 24549 | 746 | 75 | 434 |
| EOG7HTV29 | 242155 | 243615 | 1460 | 39 | 438 |
| EOG76B94T | 74635 | 75861 | 1226 | 126 | 445 |
| EOG7KX43F | 51757 | 53016 | 1259 | 66 | 460 |
| EOG7DCCQ3 | 41341 | 42498 | 1157 | 41 | 465 |
| EOG76MWV7 | 217903 | 219243 | 1340 | 15 | 466 |
| EOG7GZ0XP | 104875 | 105990 | 1115 | 107 | 467 |
| EOG7C5XWM | 78997 | 79707 | 710 | 66 | 474 |
| EOG70WF04 | 144640 | 145677 | 1037 | 30 | 476 |
| EOG7DCCQ7 | 127261 | 128832 | 1571 | 54 | 483 |
| EOG7H4RDN | 139684 | 140793 | 1109 | 37 | 492 |
| EOG7PSC1H | 82600 | 83391 | 791 | 87 | 492 |
| EOG7M3VKP | 30223 | 32004 | 1781 | 98 | 505 |
| EOG7P90TP | 28738 | 30222 | 1484 | 50 | 510 |
| EOG7H7MN1 | 43009 | 44889 | 1880 | 66 | 529 |
| EOG7M10B2 | 215755 | 217152 | 1397 | 12 | 535 |
| EOG7DP0DV | 250330 | 251592 | 1262 | 101 | 541 |
| EOG7S2CHH | 252619 | 253479 | 860 | 98 | 545 |
| EOG7PCW2Z | 228865 | 229530 | 665 | 75 | 548 |
| EOG72RZJH | 256120 | 257085 | 965 | 99 | 551 |
| EOG7PCW39 | 224230 | 225024 | 794 | 36 | 565 |
| EOG77QGGZ | 83392 | 84207 | 815 | 69 | 570 |
| EOG7P38BX | 128833 | 130260 | 1427 | 108 | 570 |
| EOG7NWHWR | 13093 | 13989 | 896 | 66 | 571 |
| EOG7CKDSD | 152767 | 153681 | 914 | 46 | 575 |
| EOG789PX7 | 161938 | 163782 | 1844 | 88 | 601 |
| EOG75JB7C | 238807 | 240138 | 1331 | 111 | 649 |
| EOG7C8T2P | 115288 | 116574 | 1286 | 66 | 764 |
| EOG7Z9H5W | 48742 | 50088 | 1346 | 31 | 776 |
| EOG7MM6S5 | 27658 | 28737 | 1079 | 68 | 776 |
| EOG735FG7 | 197359 | 202173 | 4814 | 126 | 851 |
| EOG75R2PJ | 166867 | 168576 | 1709 | 57 | 885 |

**Table S4** Pairwise genetic differentiation (mean *Fst*) among geographic populations

| **Geographic population** | **USA** | **Philippine** | **China** |
| --- | --- | --- | --- |
| **USA (51)** | - | 0.000 | 0.000 |
| **Philippine (18)** | 0.03321* | - | 0.000 |
| **China (20)** | 0.036626* | 0.0334* | - |

**Notes:** *Fst* values are below diagonal and their *P* values are above diagonal. asterisk * indicates a significance at the level of *P* < 0.05. The number in the parentheses represents the sample size in a geographic population. Samples in USA from the PT group were excluded in this analysis.

**Table S5** Analysis of molecular variance (AMOVA) associated with host plant and geography

| **Source of variation** | | **df** | **Sum of squares** | **Variance components** | **Percentage variation** | ***P* value** |  |
| --- | --- | --- | --- | --- | --- | --- | --- |
| **Host plant** | |  |  |  |  |  |  |
| Among pop. | | 9 | 488.014 | 1.13089a | 3.55155 | < 0.001 |  |
| Within pop. | | 132 | 6664.793 | 30.71126b | 96.44845 |  |  |
| **Geography** |  | | | | | | |
| Among pop. | | 2 | 414.022 | 0.11356a | 0.08445 | < 0.001 |  |
| Within pop. | | 88 | 19129.237 | 134.20554b | 99.91545 |  |  |

**Notes:** letters a and b in variance components suggest genetic variances are significantly different for individuals from among and within populations at the significant level of 0.05. pop. = population.

**Table S6** AMOVA between PT and non-PT groups

| **Source of variation** | **df** | **Sum of squares** | **Variance components** | **Percentage of variation (%)** | ***P* value** |
| --- | --- | --- | --- | --- | --- |
| **Among populations** | 1 | 602.120 | 2.85232a | 2.88795 | <0.001 |
| **Within populations** | 132 | 19267.154 | 95.91414b | 97.11205 |  |
